# Quantitative Brightness Analysis of Fluorescence Intensity Fluctuations in E. Coli

**DOI:** 10.1371/journal.pone.0130063

**Published:** 2015-06-22

**Authors:** Kwang-Ho Hur, Joachim D. Mueller

**Affiliations:** 1 School of Physics and Astronomy, University of Minnesota, Minneapolis, Minnesota, United States of America; 2 Department of Biomedical Engineering, University of Minnesota, Minneapolis, Minnesota, United States of America; Irvine, UNITED STATES

## Abstract

The brightness measured by fluorescence fluctuation spectroscopy specifies the average stoichiometry of a labeled protein in a sample. Here we extended brightness analysis, which has been mainly applied in eukaryotic cells, to prokaryotic cells with E. coli serving as a model system. The small size of the E. coli cell introduces unique challenges for applying brightness analysis that are addressed in this work. Photobleaching leads to a depletion of fluorophores and a reduction of the brightness of protein complexes. In addition, the E. coli cell and the point spread function of the instrument only partially overlap, which influences intensity fluctuations. To address these challenges we developed MSQ analysis, which is based on the mean Q-value of segmented photon count data, and combined it with the analysis of axial scans through the E. coli cell. The MSQ method recovers brightness, concentration, and diffusion time of soluble proteins in E. coli. We applied MSQ to measure the brightness of EGFP in E. coli and compared it to solution measurements. We further used MSQ analysis to determine the oligomeric state of nuclear transport factor 2 labeled with EGFP expressed in E. coli cells. The results obtained demonstrate the feasibility of quantifying the stoichiometry of proteins by brightness analysis in a prokaryotic cell.

## Introduction

Fluorescently labeled proteins produce intensity fluctuations as they pass through a small observation volume. Fluorescence correlation spectroscopy (FCS) and fluorescence fluctuation spectroscopy (FFS) exploit these fluctuations to characterize diffusional mobility, concentration, and brightness of the labeled proteins [1–3]. Because a fluorescence fluctuation experiment passively observes the sample, it provides a powerful approach to characterize the behavior of labeled proteins directly inside a living cell from the analysis of the steady-state intensity fluctuations. The brightness parameter is of special interest. It measures the average fluorescence intensity per particle and is directly related to the stoichiometry of a protein complex [2,4]. For example, monomers that associate into dimers double the brightness, because the protein complex contains two labeled fluorophores. After demonstrating the feasibility of quantifying protein interactions in a living cell by brightness analysis [2] the technique has matured into a widely used tool for identifying intracellular protein association in eukaryotic cells [3,5–7].

This work extends brightness analysis to prokaryotes, using the bacterium E. coli as a model system. The volume of a typical bacterial cell is on the order of a femtoliter, while a typical mammalian cell has a volume of a few picoliters. This reduction in volume introduces significant challenges. First, the size of the bacterium is smaller than the point spread function (PSF) of the optical microscope. The incomplete overlap between PSF and sample changes the amplitude distribution of the fluorescence intensity fluctuations and therefore distorts the brightness [6,7]. Second, because the excitation beam illuminates a large part of the bacterial volume, photobleaching results in a noticeable decrease in the number of active fluorophores. We refer to this cumulative decrease in the population of fluorescent molecules as photodepletion, which has been discussed in more detail recently [8]. Photodepletion is not accounted for by traditional FFS analysis and can cause spurious results [8].

Z-scan FFS and segmented brightness analysis (SBA) were independently developed to account for incomplete PSF overlap and photodepletion in eukaryotic cells, respectively [6,8], but we found that these methods were not adequate for experiments on prokaryotes. In response, we developed in this work mean segmented Q-value (MSQ) analysis and combined it with a modified z-scan FFS theory to evaluate the brightness of labeled proteins in E.coli. We present a rigorous derivation of MSQ analysis. While the derivation is lengthy and proceeds through a number of intermediate steps, the final result is simple and elegant. We demonstrate that MSQ analysis coupled with z-scan FFS recovers not only brightness but also the concentration and diffusion time. We first applied the technique to recover the brightness of enhanced green fluorescent protein (EGFP) in mammalian, yeast, and E. coli cells. Next, EGFP-labeled nuclear transport factor 2 (NTF2), which has been shown to be dimeric in U2OS cells [6,9], was measured in E. coli, to evaluate the potential of our new algorithm to determine the oligomeric state of a soluble protein in a bacterial cell. We found that the performance of MSQ analysis in E. coli cells is comparable to established brightness analysis methods in mammalian cells.

## Materials and Methods

### Instrumentation

Samples were measured on a home-built two-photon microscope based on an Axiovert 200 microscope (Zeiss, Thornwood, NY) interfaced with a Ti:Sapphire laser (Tsunami, Spectra Physics, Mountain View, CA) with an excitation wavelength of 1000 nm and a power of ~1 mW. The fluorescence was collected with a 63x C-Apochromat water immersion objective lens (NA = 1.2, Zeiss) and registered by a photodetector (HPM-100-40, Becker & Hickl, Berlin, Germany) connected to a photon counting acquisition card (ISS, Champaign, IL), which recorded data with a frequency of 20 kHz. A dichroic mirror (Chroma Technology, Rockingham, VT) served to separate excitation and emission light. The z-scan was performed by moving the stage (PZ2000 piezo stage, ASI, Eugene, OR) along the direction of the beam path [6]. The stage was driven by a voltage signal from an arbitrary waveform generator (33250A, Agilient Technologies, Santa Clara, CA). The signal waveform was a linear ramp function with a frequency of 0.1 Hz and a peak-to-peak amplitude of 0.8 V, which corresponds to 8.04 μm of axial travel. The z-scan intensity profile was sampled at 20 kHz.

### Expression vectors

EGFP was amplified from the pEGFP-C1 plasmid (Clontech, Mountain View, CA) with a 5’ primer that encodes a BamHI restriction site and a 3’ primer that encodes an XhoI site. The product was cloned into the pRSET-B vector (Invitrogen, Carlsbad, CA), which is referred to as pB-G and serves as the E. coli expression vector. NTF2 was amplified from human NTF2 expression vector (Genbank accession number: BC002348) with a 5’ primer that encodes an XhoI restriction site and a 3’ primer that encodes an EcoRI site. The result was cloned into the EcoRI/HindIII site of pB-G. Expression vectors for yeast and U2OS cells have been described previously [8].

### Sample preparations

The competent E. coli strain BL21(DE3)pLysS (Promega, Madison, WI) was used in this study. E. coli cells carrying either the EGFP or NTF2-EGFP vector were cultured overnight at ~30 C° in Lysogeny broth (LB) medium with 1 mM ampicillin. The medium was diluted to 0.2–0.3 OD_600nm_ with fresh LB medium the next morning. After growing to 0.6–0.8 OD_600nm_ the medium was centrifuged at 6000 g for ~10 s. After removing the old medium the cells were resuspended with fresh LB medium and mixed with low-melting point agarose dissolved in PBS medium at ~32 C°. A volume of 0.5 μl of the 1% agarose/medium mixture was transferred to a microscope slide and covered by a coverslip, which was gently pressed to achieve a layer thickness of ~1 μm between the microscope slide and the coverslip. This process resulted in an orientation of E. coli cells parallel to the glass interface. The slide borders were sealed with nail polish. The preparation of yeast and U2OS samples has been described elsewhere [8]. In addition, EGFP was purified as reported [10] and dissolved in Biacore's HBS-EP buffer (10 mM HEPES pH 7.4, 150 mM NaCl, 3 mM EDTA, 0.005% v/v Surfactant P20) for solution measurements.

### Measurement Protocol and Analysis

The FFS experiments in U2OS and yeast cells were performed as previously described [2,8,11]. For experiments on E. coli the bacteria were first identified in bright-field illumination using a CCD camera. The focal point of the two-photon beam was aligned with the geometric center of the imaged E. coli cell, followed by a z-scan at a reduced power of ~0.3 mW, which ensured that photodepletion was negligible during the scan. Before performing the FFS measurement, the beam position was moved axially until the fluorescent intensity was maximized, which corresponds to a focus at the midpoint of the E. coli cell. The beam power was then switched to ~1 mW to collect photon counts for the FFS experiment. The analysis of the FFS experiments and the z-scan intensity profiles is described in the Results section. Artifacts due to undersampling of fluctuations are negligible, since data were sampled faster than the residence time of the labeled protein. We also performed solution measurements of EGFP to provide a reference brightness *λ*_*EGFP*_ or reference Q-factor *Q*_*EGFP*_ for the cell experiments. These measurements were taken with the focus ~10 μm into the solution to ensure that the PSF is completely embedded in the solution. All data were analyzed with programs written in IDL 8.3 (Research Systems, Boulder, CO).

### Z-scan Calibration of PSF

A modified squared Gaussian-Lorentzian (mGL) model,

$$PSF\left(x,y,z\right)={\left(\frac{z_{0}^{2}}{z_{0}^{2}+z^{2}}\right)}^{\left(1+\eta \right)}\text{exp}\left(-\frac{4z_{0}^{2}}{w_{0}^{2}}\frac{\left(x^{2}+y^{2}\right)}{z_{0}^{2}+z^{2}}\right),$$ (1)

provides a good approximation of the PSF of our two-photon microscope [6,7]. A z-scan calibration procedure was performed as described previously [6] to determine the free parameters of our model. The calibration resulted in *z*_0_ = 0.86 ± 0.08 μm, *η* = 2.20 ± 0.22, and *ω*_0_ = 0.43 ± 0.05 μm, where *ω*_0_ and *z*_0_ describe the radial and axial beam waist, while *η* characterizes the axial decay shape of the PSF. The mGL PSF volume is determined by [6]

$$V_{\infty }=\left(\frac{1}{4}\pi w_{0}^{2}z_{0}\right)\frac{\sqrt{\pi }\;\Gamma \left(\eta -\frac{1}{2}\right)}{\Gamma \left(\eta \right)},$$ (2)

which yields 0.18 fl for the calibrated parameters.

## Results

Before measuring in E. coli we performed a control FFS experiment in the nucleus of U2OS cells expressing EGFP by recording the sequence of photon counts *k*_*i*_ with a sampling time *T* of 50 μs. Each photon count *k*_*i*_ is related to the photon count rate by *F*(*iT*) = *k*_*i*_/*T*, which is traditionally also referred to as the fluorescence intensity as discussed in more detail elsewhere [12]. Because the intensity is constant (S1 Fig), conventional FFS theory, which assumes a stationary fluorescence signal, applies. We used Mandel’s Q-parameter to determine the brightness *λ* of the sample from the photon counts [13],

$$Q=\frac{\kappa_{\left[2\right]}}{\kappa_{\left[1\right]}}=\frac{\langle \Delta k^{2}\rangle -\langle k\rangle }{\langle k\rangle }=\frac{\langle \Delta F^{2}\rangle }{\langle F\rangle }T=\gamma_{2}\lambda T,$$ (3)

where *γ*_2_ is the PSF gamma factor [12,14]. This equation summarizes important relations of *Q* that hold in conventional FFS theory. The population mean 〈*k*〉 and variance 〈Δ*k* ^2^〉 of the recorded photon counts *k*_*i*_ are linked to the first *κ*_[1]_ = 〈*k*〉 and second *κ*_[2]_ = 〈Δ*k* ^2^〉−〈*k*〉 factorial cumulant of *k*_*i*_ [12]. The mean and variance of the fluorescence intensity are given by 〈*F*〉 = 〈*k*〉/*T* and 〈Δ*F* ^2^〉 = (〈Δ*k* ^2^〉−〈*k*〉)/*T* ^2^. By using Eq 3 we determined *Q* = 0.018 for EGFP in the U2OS cell, which corresponds to a brightness of *λ* = 1.28 kcps. We typically convert *λ* or *Q* into a normalized value

$$b=\frac{Q}{Q_{EGFP}}=\frac{\lambda }{\lambda_{EGFP}}$$ (4)

by taking the ratio with the reference brightness *λ*_*EGFP*_ or Q-value *Q*_*EGFP*_ of the label EGFP, which were determined from an independent solution measurements of EGFP. The normalized brightness reflects the average stoichiometry of the fluorescently labeled protein. In other words, a monomeric protein corresponds to *b* = 1, while a dimeric protein results in *b* = 2. We measured a Q-value *Q*_*EGFP*_ of 0.019 and determined a normalized brightness of *b* = 0.95, which is consistent with a monomeric EGFP in cells, since the typical uncertainty of *b* measured in mammalian cells is ~10% [15,16]. Eq 4 assumes that both Q-values are measured with the same PSF and comparable overlap between PSF and sample as will be discussed later.

**Fig 1:**
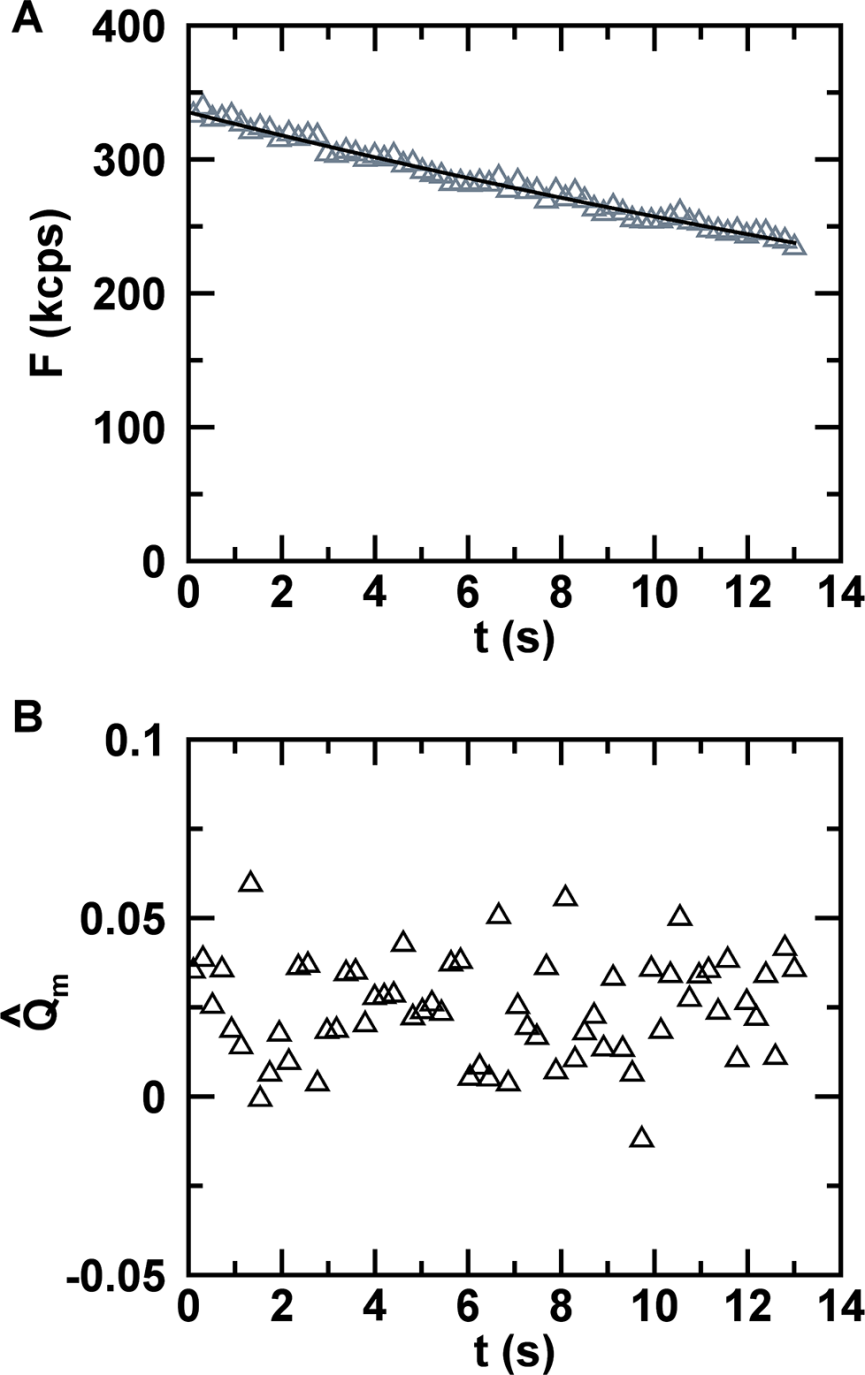
Fluorescence from EGFP in E. coli cell. (A) Fluorescence intensity (triangles) decays with time as a result of photodepletion. The fit (solid line) to an exponential decay function recovered an initial intensity *F*_0_ = 336 kcps and a depletion rate coefficient *k*_*D*_ = 0.026 s^-1^. (B) Segmented Q-values $\hat{Q_{m}}\left(T_{S}\right)$ for a segment time of *T*_*S*_ = 0.2 s with an average value of 0.025.

**Fig 2:**
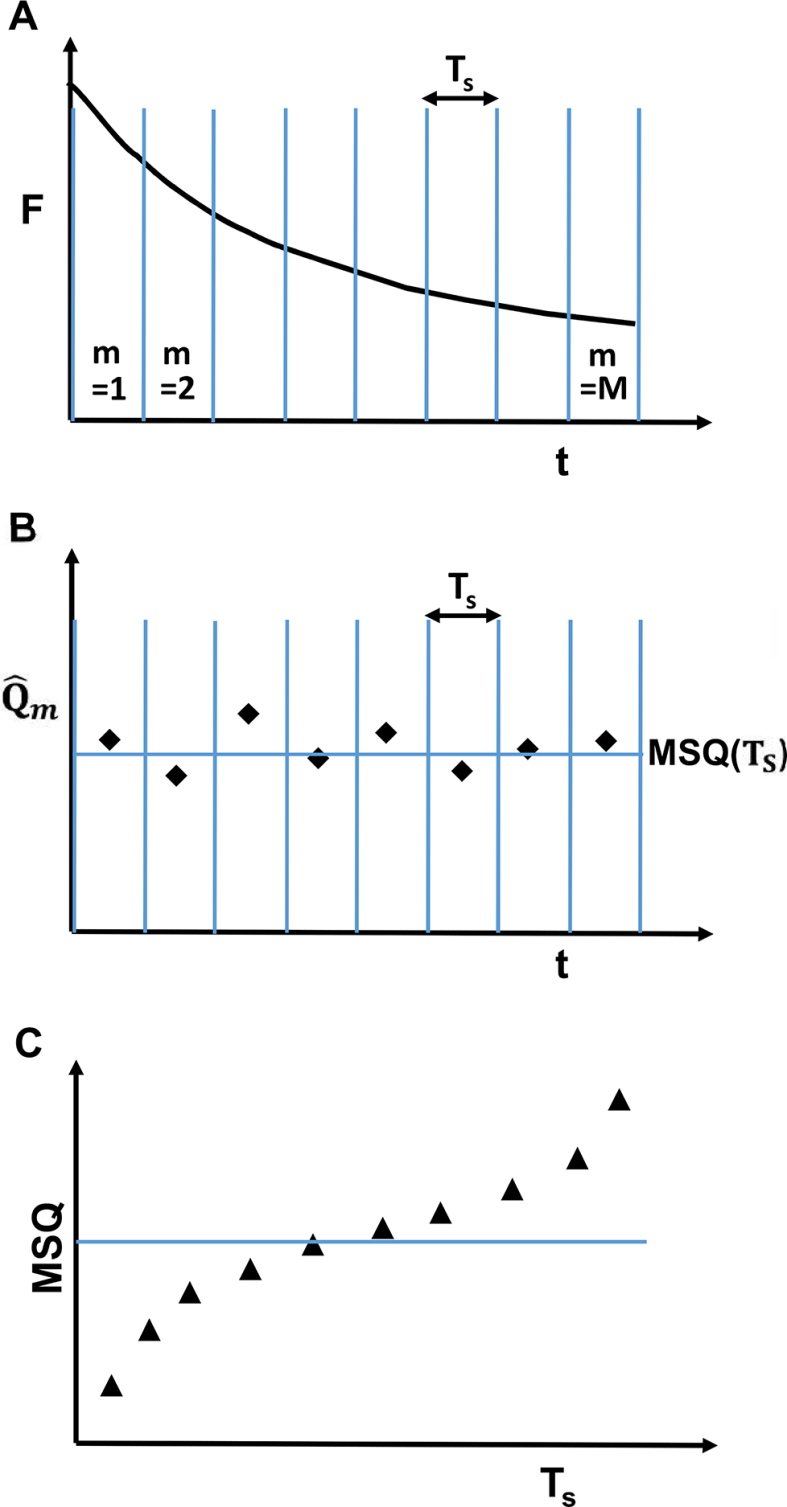
Schematic representation of MSQ analysis procedure. (A) The decaying fluorescence intensity trace is divided into M segments. Each segment has a length of *T*_*S*_. (B) The Q-value $\hat{Q_{m}}\left(T_{S}\right)$ is calculated from the photon count data of each segment, followed by the calculation of the mean of the segmented Q-values MSQ(*T*_*S*_). (C) The above steps are repeated for different segment lengths to calculate MSQ as a function of *T*_*S*_. Conventional FFS theory predicts that MSQ is independent of the segment length (solid line). The presence of photodepletion and estimator bias introduces curvature into the MSQ-curve (triangles).

When we performed an FFS experiment in E. coli expressing EGFP, the fluorescence intensity *F*(*t*) was not stationary as in the U2OS cell, but decayed exponentially (Fig 1A) from an initial intensity *F*_0_,

$$F(t)=F_{0}\;\text{exp}\left(-k_{D}t\right),$$ (5)

because photobleaching within the very small volume of the bacterium leads to photodepletion with a rate coefficient *k*_*D*_. Because the decaying signal is non-stationary, applying Eq 3, which is based on conventional FFS theory, can result in strongly biased brightness values [8]. SBA theory was introduced to circumvent this bias by dividing the intensity trace into segments (Fig 2A) short enough that the intensity decay per segment is negligible [8]. This process leads to quasistationary data within a segment provided that the segment time *T*_*S*_ does not exceed a limit *T*_*S*,limit_, which is determined by SBA theory from the intensity decay curve. SBA calculates the unbiased brightness *λ* from the segmented FFS data as previously demonstrated [8]. Applying SBA analysis to the E. coli data of Fig 1A determined a very short limit (*T*_*S*,limit_ = 0.2 s), which reflects the relatively fast intensity decay within the bacterium. To test the SBA model for such short data sections, we calculated the brightness for segment times of 0.2 s, 0.05 s, and 0.025 s and recovered 1.78, 1.49, and 1.21 kcps, respectively. Instead of recovering the same value as expected from SBA theory, we observed a decrease in brightness at shorter segment times. This result demonstrated that SBA analysis is not suitable for E. coli samples.

Thus, we set out to develop a robust analysis method that is equally applicable in large eukaryotic and small prokaryotic cells. We define a few quantities used throughout the paper. The experimental data are divided into *M* = *T*_DAQ_/*T*_*S*_ segments with *T*_DAQ_ being the total data acquisition time and *T*_*S*_ representing the segment time (Fig 2A). The m-th segment defined by the time interval [(*m*−1)*T*_*S*_, *mT*_*S*_] contains *N* = *T*_*S*_/*T* sampled photon count events *k*_*m*,*i*_ sampled with a time interval *T*. The unbiased estimator of the first two photon count moments, $\hat{k_{m}}\equiv N^{-1}\sum_{i=1}^{N}k_{m,i}$ and $\hat{k_{m}^{2}}\equiv N^{-1}\sum_{i=1}^{N}k_{m,i}^{2}$, were used to construct an estimator of Q for the m-th segment based on Eq 3

$$\hat{Q_{m}}\left(T_{S}\right)=\frac{\hat{\Delta k_{m}^{2}}-\hat{k_{m}}}{\hat{k_{m}}}=\frac{\hat{k_{m}^{2}}}{\hat{k_{m}}}-\hat{k_{m}}-1,$$ (6)

with an estimator of the variance defined by $\hat{\Delta k_{m}^{2}}\equiv N^{-1}\sum_{i=1}^{N}{\left(k_{m,i}-\hat{k_{m}}\right)}^{2}$. Applying Eq 6 determines the Q-value for each segment as illustrated in Fig 2B. The experimental segmented Q-values for the E.coli data depicted in Fig 1A are shown in Fig 1B for *T*_*S*_ = 0.2 s. We also efine the average of the Q-estimator over all segments,

$$\text{MSQ}\left(T_{S}\right)=M^{-1}\sum_{m=1}^{M}\hat{Q_{m}}\left(T_{S}\right),$$ (7)

which we refer to as the mean of the segmented Q-values (MSQ). The MSQ-curve is constructed by calculating Eq 7 for many different segment times and will be the centerpiece of the new analysis method introduced here (Fig 2C). Conventional FFS as described by Eq 3 predicts a MSQ-curve that is independent of the segment length *T*_*S*_ (solid line, Fig 2C). Any observed changes of MSQ with *T*_*S*_ reflect the presence of an artifact that needs to be accounted for. In the following, we will present experimental MSQ-curves and develop the theory to model the data.

**Fig 3:**
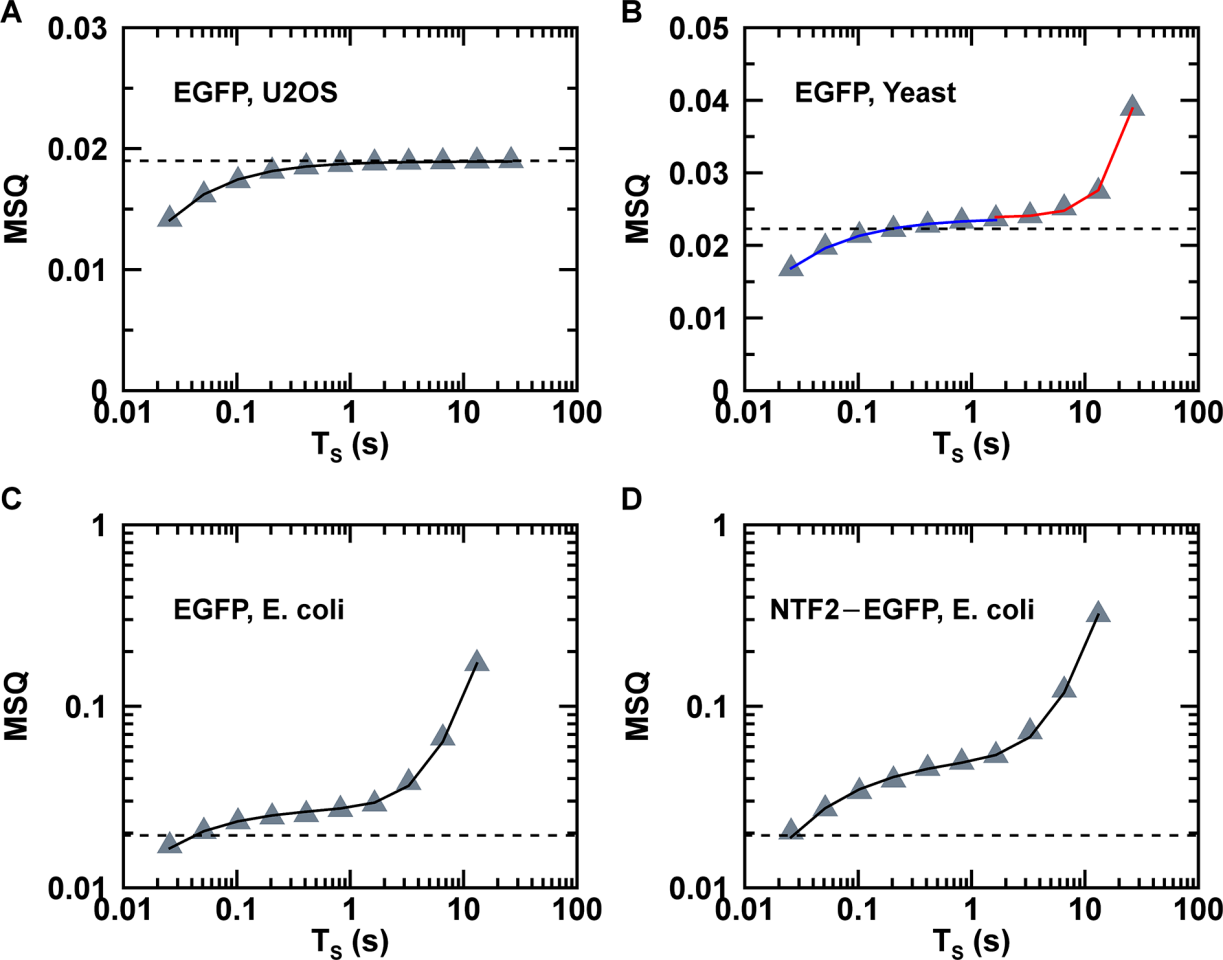
MSQ curves. (A) MSQ-curve (triangles) for EGFP in U2OS cell and fit (solid line) to MSQ model with *Q* = 0.0193 and a diffusion time of 0.8 ms. (B) MSQ-curve (triangles) of EGFP in yeast cell. Fit (blue line) of data with *T*_*S*_ < 1.6 s to Eq 8 yielded *Q* = 0.0237 and *τ*_*D*_ = 1.2 ms. Fit (red line) of data with *T*_*S*_ > 1.6 s to Eq 12 determined *Q* = 0.0238 and *k*_*D*_ = 4.46 × 10^−3^ s^-1^. (C) MSQ-curve (triangles) of EGFP in E.coli cell and fit (solid line) to Eq 14 with *Q* = 0.028, *τ*_*D*_ = 2.7 ms and *k*_*D*_ = 2.7 × 10^−2^ s^-1^. (D) MSQ-curve (triangles) of NTF2-EGFP in E.coli cell and fit (solid line) to Eq 14 with *n* = 2.1, *τ*_*D*_ = 10 ms and *k*_*D*_ = 5.3 × 10^−2^ s^-1^. The dashed line in each panel represents the reference Q-value of EGFP in solution, which was measured at the same power as the corresponding MSQ data.

We calculated the MSQ-curve for FFS data of EGFP measured in U2OS, yeast and E. coli (Fig 3A, 3B and 3C) and observed a clear dependence of MSQ on *T*_*S*_. Similarly, repeating the process on data from E. coli expressing NTF2-EGFP resulted in a pronounced dependence of MSQ on *T*_*S*_ (Fig 3D). The MSQ-curve from the U2OS cell expressing EGFP (Fig 3A) comes closest to the ideal behavior. The MSQ-factor stays essentially constant for *T*_*S*_ > 1s and only appreciably drops for *T*_*S*_ less than ~0.4s. Performing the same experiment in yeast cells resulted in a MSQ-curve (Fig 3B) with a similar decline at short segment times as seen with the U2OS cells. However, unlike the U2OS cells, the MSQ-curve rises at long segment times, indicating an apparent increase in brightness. We previously demonstrated that photobleaching, which leads to a depletion of the fluorophores within the small volume of the yeast cell, introduces artificially inflated brightness values [8]. Because the volume of a U2OS cell vastly exceeds that of yeast, the same photobleaching process results in an entirely negligible depletion of the fluorophore population in the larger cell [8]. The MSQ-curves for E. coli (Fig 3C and 3D) are graphed with a logarithmic y-axis and display the same general behavior as observed for yeast, only more pronounced.

A common feature of all experimental MSQ-curves is the observed decrease at short segment times. We suspected that estimator bias is responsible for this effect, because it also affects the autocorrelation function [17]. The Q-estimator of Eq 6 involves the ratio of two unbiased estimators ($\hat{k_{m}^{2}}$ and $\hat{k_{m}}$) and therefore is only asymptotically unbiased [18]. We started with the definition of Eq 6 and derived the expectation value of the MSQ function accounting for the estimator bias (see section A of S1 File),

$${\text{MSQ}}_{EB}\left(T_{S}\right)=Q-\frac{1}{N}-Q\frac{B_{2}\left(NT,\tau_{D}\right)}{{\left(NT\right)}^{2}},$$ (8)

where *B*_2_(*T*, *τ*_*D*_) represents the second-order binning function [12,14] with *τ*_*D*_ as the diffusion time. Fitting the MSQ-curve from the U2OS cell to Eq 8 leads to a good representation of the experimental data with *τ*_*D*_ = 0.80 ms and *Q* = 0.019 (Fig 3A). The fitted diffusion time is in good agreement with the diffusion time of 0.72 ms determined by a fit of the autocorrelation function of the U2OS data. The fitted Q-value matches the calibration value (*Q*_*EGFP*_ = 0.019) for EGFP in solution.

**Fig 4:**
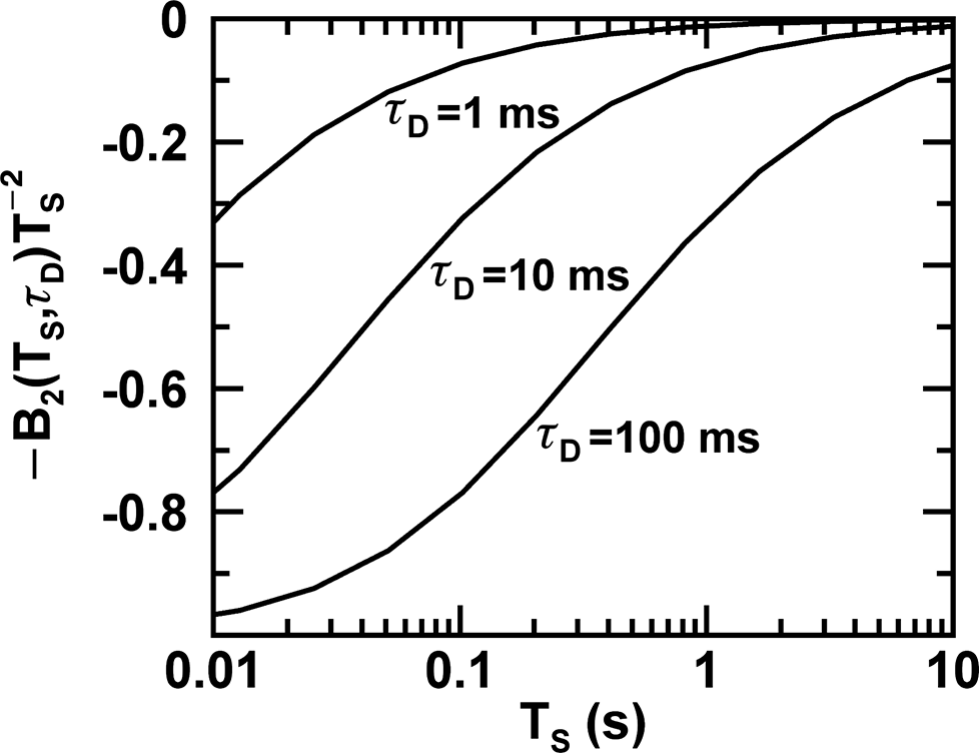
Relative bias in the Q-value introduced by diffusion. Correlations in the photon counts introduced by the diffusion time *τ*_*D*_ give rise to a relative bias in the Q-estimator that depends on the segment period *T*_*S*_. The relative bias for diffusion times of 1 ms, 10 ms, and 100 ms is shown.

The relative bias between the MSQ-value and the true Q-value depends on the second and third term of Eq 8. The influence of the second term on the MSQ-value is mostly negligible, since *N* ≥ 500 for all data shown in Fig 3, which results in a maximum relative bias of ~10% at the shortest segment length. The third term, which arises from the correlation in the photon counts, becomes more important as the ratio *T*_*S*_/*τ*_*D*_ decreases. The relative bias in MSQ due to the third term is $-B_{2}\left(T_{S},\tau_{D}\right)/T_{S}^{2}$ and exceeds 10% once *T*_*S*_ < 50*τ*_*D*_ (Fig 4). This result demonstrates that slow diffusing species are more prone to estimator bias than fast diffusing species. The bias in the MSQ decreases with increasing *T*_*S*_ and disappears in the limit *T*_*S*_ → ∞, which demonstrates that Eq 8 describes an asymptotically unbiased estimator.

Next we analyzed photodepletion following a previously discussed approach [8] to model the increase in the MSQ-curve at long segment times (Fig 3B and 3C). Consider an unbiased estimator $\hat{g_{m}}$ of the form $N^{-1}\sum_{i=1}^{N}g_{m,i}$. A non-stationary signal introduces a time-dependent population mean 〈*g*_*m*_ (*t*)〉. Since $\hat{g_{m}}$ involves a summation over the m-th segment of duration *T*_*S*_, the expectation value $E\hat{g_{m}}\equiv \langle g_{m}\rangle$ represents the time-average of 〈*g*_*m*_ (*t*)〉 over the segment,

$$\bar{\langle g_{m}\rangle }\equiv N^{-1}\sum_{i=1}^{N}\langle g_{m}\left(t_{i}\right)\rangle \approx \frac{1}{T_{S}}\int_{(m-1)T_{S}}^{mT_{S}}\langle g(t)\rangle dt,$$ (9)

where we approximated the summation by a time integral and *t*_*i*_ = (*m*−1)*T*_*S*_ + *iT*. We are using the convention that a bar over a variable specifies the time average of that variable over one segment. By applying Eq 9 to the estimators $\hat{k_{m}}$ and $\hat{\Delta k_{m}^{2}}$ we derived an expression for the expectation value of the Q-estimator $E\hat{Q_{m}}$ using time-averaged cumulants (see section B of S1 File),

$$E\hat{Q_{m}}=\frac{\bar{\kappa_{\left[2\right]m}}+\bar{\kappa_{\left[1\right]m}^{2}}-{\left(\bar{\kappa_{\left[1\right]m}}\right)}^{2}}{\bar{\kappa_{\left[1\right]m}}}.$$ (10)

Eq 10 can only be applied to long segment times, because estimator bias has been ignored for now.

We evaluate Eq 10 for the special case of a monomeric protein, such as EGFP, in the presence of photodepletion with rate coefficient *k*_*D*_ and a photobleaching reaction, *F* → *D*, that converts the fluorescent state *F* into a non-fluorescent state *D*. The first two time-dependent cumulants of this model have been derived in previous work [8],

$$\begin{array}{l} \kappa_{\left[1\right]}\left(t\right)=\lambda TN_{0}e^{-k_{D}t}\;,\\ \kappa_{\left[2\right]}\left(t\right)=\gamma_{2}\lambda T\kappa_{\left[1\right]}\left(t\right), \end{array}$$ (11)

where *N*_0_ is the initial average number of fluorescence proteins in the observation volume, which is related to the initial fluorescence intensity by *F*_0_ = *λN*_0_. We calculated the time-averaged expressions for $\bar{\kappa_{\left[2\right]m}}$, $\bar{\kappa_{\left[1\right]m}^{2}}$ and $\bar{\kappa_{\left[1\right]m}}$ based on Eq 11, which were inserted into Eq 10. Next, we determined a model for the MSQ curve in the presence of photodepletion by averaging over all segments, ${\text{MSQ}}_{PD}\left(T_{S}\right)=M^{-1}\sum_{m=1}^{M}E\hat{Q_{m}}\left(T_{S}\right)$,

$${\text{MSQ}}_{PD}\left(T_{S}\right)=A\left(Q_{1},n\right)+F_{0}T\frac{1-{\left(1-\Delta f_{D}\right)}^{M}}{M\Delta f_{D}}\left(\frac{2-\Delta f_{D}}{2}+\frac{\Delta f_{D}}{\text{ln}\left(1-\Delta f_{D}\right)}\right).$$ (12)

A detailed derivation of this equation is found in section C of S1 File. The equation depends on the number of segments *M* = *T*_*DAQ*_/*T*_*S*_, the initial fluorescence intensity *F*_0_, and the depletion fraction,

$$\Delta f_{D}\left(T_{S}\right)=1-\text{exp}\left(-k_{D}T_{S}\right).$$ (13)

The depletion fraction Δ*f*_*D*_ describes the fractional decrease of the fluorescence intensity from the beginning to the end of a segment, Δ*f*_*D*_ = 1−*F*(*mT*_*S*_)/*F*((*m*−1)*T*_*S*_), which for an exponential decay with the depletion rate constant *k*_*D*_ reduces to Eq 13. The function A(*Q*_1_, *n*), which depends on the Q-factor *Q*_1_ for a monomer and the stoichiometry *n* of the protein sample, will be discussed in more detail later. From here on we have to distinguish carefully between the Q-factor *Q*_1_ of a monomer and the Q-factor *Q* = *nQ*_1_ of an n-mer. For the special case of a monomer (*n* = 1) considered for now, the function reduces to A(*Q*_1_, 1) = *Q*_1_.

We applied the above theory to the MSQ-curve of EGFP in yeast (Fig 3B). SBA analysis of the yeast data indicated that photodepletion effects are only significant for *T*_*S*_ > 1.6 s. Thus, we fit the MSQ-curve for *T*_*S*_ > 1.6 s to Eq 12 with A(*Q*_1_, 1) = *Q*_1_. The only fit parameters were *Q*_1_ and the depletion rate coefficient *k*_*D*_ of Eq 13. The number of segments *M* for each *T*_*S*_ is known and the initial intensity *F*_0_ was determined from the intensity decay curve of the yeast data. The experimental MSQ-curve for *T*_*S*_ > 1.6 s is reproduced by the fit (red line, Fig 3B) to the MSQ_*PD*_ model with *Q*_1_ = 0.0238. The fitted depletion rate coefficient of 0.0045 s^-1^ is in good agreement with the value of 0.0044 s^-1^ recovered by an independent fit of the intensity decay (S2 Fig). Next, we modeled the experimental MSQ-curve for *T*_*S*_ < 1.6 s. Because we expected that this part of the curve is only influenced by estimator bias, we modeled it using Eq 8. The fit (blue line, Fig 3B) to this equation faithfully describes the MSQ-curve at small segment times with a fitted Q-value of 0.0237. Both fitted Q-values are in close agreement. Comparing both Q-values with the in vitro calibrated monomeric Q-value (*Q*_*EGFP*,∞_ = 0.0223, Fig 3B) results in a normalized brightness of 1.06, which is consistent with a monomeric protein.

The yeast data demonstrated that photodepletion bias and estimator bias can affect different domains of the MSQ-curve. Short segment lengths suffer from estimator bias, while long segment lengths are affected by photodepletion bias. The plateau in the MSQ curve around *T*_*S*_ ≈ 1 s separates these two domains. Segment times that correspond to the plateau region are essentially free from either artifact, which validates our previous SBA analysis of yeast FFS data [8]. The MSQ-curve for E. coli (Fig 3C), on the other hand, lacks the plateau region found for yeast, which implies that no region of the MSQ-curve is free of bias. This observation further implies that a range of segment lengths exists where both biases are present simultaneously. Thus, modeling of the MSQ-curve requires the following formula that combines both photodepletion and estimator bias (see section D of S1 File),

$$\text{MSQ}\left(T_{S}\right)=\text{A}\left(Q_{1},n\right)+F_{0}T\frac{1-{\left(1-\Delta f_{D}\right)}^{M}}{M\Delta f_{D}}\left(\frac{2-\Delta f_{D}}{2}+\frac{\Delta f_{D}}{\text{ln}\left(1-\Delta f_{D}\right)}\right)-\frac{1}{N}-\text{A}\left(Q_{1},n\right)\frac{B_{2}\left(T_{S},\tau_{D}\right)}{T_{S}^{2}}.$$ (14)

The first two terms are identical to Eq 12 and capture the influence of photodepletion. The next two terms describe the estimator bias and are similar to Eq 8, the only difference being the last term where *Q* has been replaced by A(*Q*_1_, *n*). We fit the entire MSQ-curve for E. coli expressing EGFP to Eq 14 with A(*Q*_1_, 1) = *Q*_1_, since EGFP is a monomeric protein. The only free fit parameters were *k*_*D*_, *τ*_*D*_ and *Q*_1_, since Δ*f*_*D*_ = 1−exp(−*k*_*D*_ *T*_*S*_), *M* = *T*_*DAQ*_/*T*_*S*_, and *N* = *T*_*S*_/*T*. The initial intensity *F*_0_ was determined by a fit of the intensity decay curve (Fig 1A). Applying Eq 14 to the MSQ data of E. coli results in a fit (solid line, Fig 3C) that closely matches the experimental curve. However, the recovered monomeric Q-value (*Q*_1_ = 0.028) was significantly higher than the calibration Q-value of EGFP (*Q*_*EGFP*_ = 0.019) measured in solution. This result implies a normalized brightness of 1.47, which is inconsistent with a monomeric sample.

This apparent contradiction is caused by differences in the overlap between the sample and the PSF volume [6,7]. The solution measurements of EGFP were taken with the focus sufficiently deep in the solution, so that the entire PSF volume is embedded in the sample. This situation mimics an infinite sample reservoir and is the standard condition assumed in traditional FFS analysis. These differences in overlap prompted us to distinguish from now on between different sample geometries. The subscript ∞ is used to mark properties that are measured or calculated for the infinite sample geometry. Thus, EGFP with brightness *λ*_*EGFP*_ measured in solution leads to a Q-factor *Q*_*EGFP*,∞_ = *γ*_2,∞_ *λ*_*EGFP*_ *T* with *γ*_2,∞_ describing the gamma factor of the entire PSF. The geometry of E. coli can be approximated by a cylinder with diameter of ~1 μm, which is too small to enclose the entire PSF volume. This incomplete overlap between bacterium and the PSF influences the measured Q-parameter [6]. FFS parameters that are measured or calculated for the cylindrical geometry of E. coli are identified by the subscript *cyl*. EGFP measured in E.coli leads to a Q-factor *Q*_*EGFP*, *cyl*_ = *γ*_2,*cyl*_ *λ*_*EGFP*_ *T* with *γ*_2,*cyl*_ describing the gamma factor of the PSF that overlaps with the sample. Thus samples with the same brightness *λ*_*EGFP*_ measured in two different sample geometries result in different Q-factors. Calculating the normalized brightness by Eq 4 implicitly assumes that the Q-factors are taken with the same overlap between sample and PSF. Thus, we need to convert the solution Q-factor *Q*_*EGFP*,∞_ of EGFP into the equivalent Q-factor *Q*_*EGFP*,*cyl*_ for E.coli before employing Eq 4. The equations *Q*_*EGFP*,∞_ = *γ*_2,∞_ *λ*_*EGFP*_ *T* and *Q*_*EGFP*,*cyl*_ = *γ*_2,*cyl*_ *λ*_*EGFP*_ *T* relate both Q-factors by *Q*_*EGFP*,*cyl*_ = *Q*_*EGFP*,∞_ *γ*_2,*cyl*_/*γ*_2,∞_. However, this procedure requires knowledge of the gamma factor ratio, which can be determined by z-scan FFS [6].

**Fig 5:**
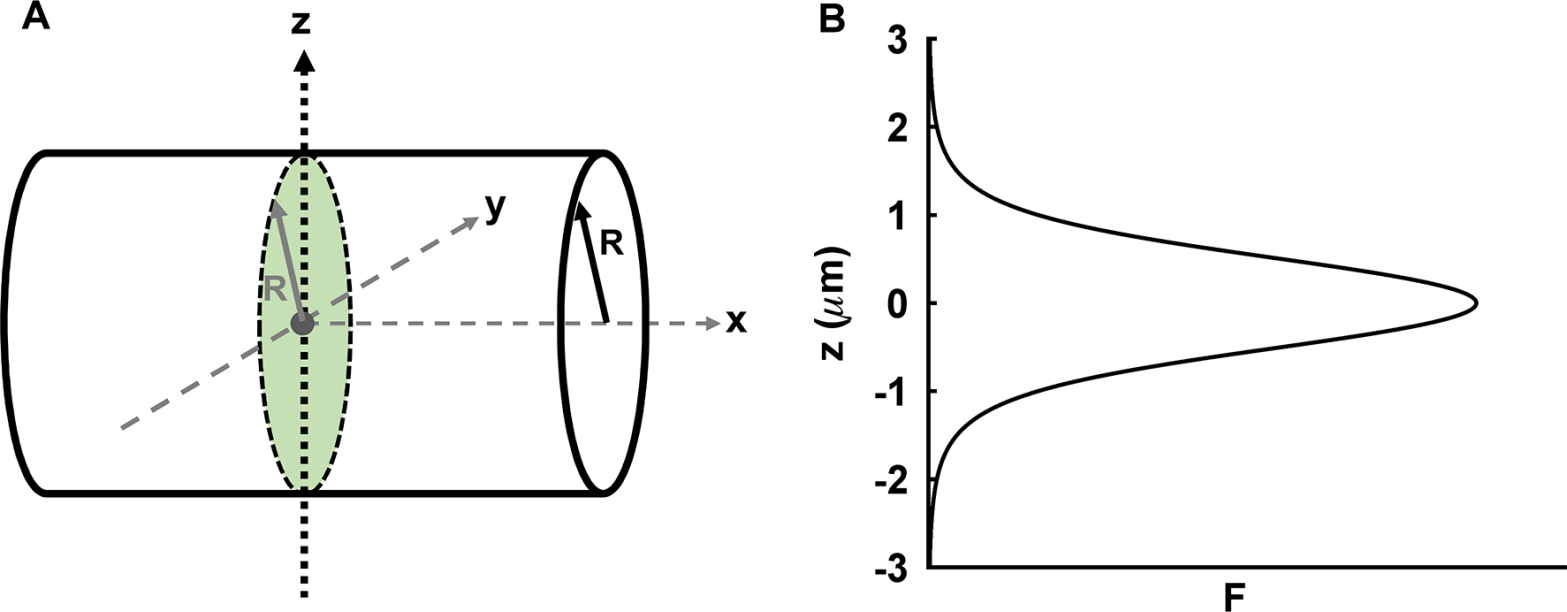
Schematic illustration of z-scan for E. coli experiments. (A) Approximation of E. coli geometry by a cylinder with radius *R*. The scan axis z passes through the geometric center of the cylinder. (B) Z-scan fluorescence intensity profile of cylinder is modeled by Eq 18.

We originally developed z-scan FFS based on earlier work by the Hof group [19] to correctly determine the brightness of thin layers, such as a thin cytoplasmic slab [6]. The fluorescence intensity profile of a z-scan through the sample determines the sample geometry, which is then used to identify the correct gamma factor for FFS experiments [6]. We followed the same approach and performed an axial scan of the PSF through the E. coli bacterium with the scan trajectory perpendicular to the rotation axis of the cylinder. The scan passes through the geometric center (Fig 5A) and generates a z-scan intensity profile (Fig 5B). Previous z-scan analysis of the intensity profile accounted for the finite sample size only along the z-direction, which for E. coli is no longer sufficient, because the finite width of the bacterium is comparable to the size of the PSF in the y direction. The length of the bacterium is sufficiently large that its finite size is not a concern. Thus, we modeled the bacterium as a cylinder of radius *R* assuming for simplicity an infinite length along the x-direction. The cylinder is defined by the set

$$V_{cyl}\left(R\right)=\left\{\left(x\prime ,y\prime ,z\prime \right)|x\prime \in \left(-\infty ,\infty \right),y\prime \in \left(-\sigma ,\sigma \right),z\prime \in \left(-R,R\right)\right\}$$ (15)

with $\sigma =\sqrt{R^{2}-{z\prime }^{2}}$. Using a coordinate system with the origin placed at the geometric center (Fig 5A), we define the effective PSF volume of order *r* by

$$V_{\text{eff}}^{\left(r\right)}\left(R,z\right)=\int_{V_{cyl}\left(R\right)}{\text{PSF}}^{r}\;\left(x\prime ,y\prime ,z\prime -z\right)dr\prime .$$ (16)

Eq 16 describes the volume overlap of PSF raised to the r-th power with the cylindrical sample volume, where the PSF is located at position *z* with respect to the geometric center along the z-axis. Evaluating Eq 16 for the modified-Gaussian Lorentzian PSF (Eq 1) results in

$$V_{\text{eff}}^{\left(r\right)}\left(R,z\right)=\frac{\pi \omega_{0}{}^{2}z_{0}}{4r}\int_{-\hat{R}}^{\hat{R}}{\left(1+{\left(\hat{z}\prime -\hat{z}\right)}^{2}\right)}^{1-r\left(1+\eta \right)}\;\text{Erf}\left(\phi_{r}(\hat{z})\right)d\hat{z}\prime$$ (17)

with $\hat{z}=z/z_{0}$, $\hat{R}=R/z_{0}$, $\phi_{r}\left(\hat{z}\right)=2\sqrt{r}\alpha \sqrt{\frac{{\hat{R}}^{2}-{\hat{z}}^{2}}{1+\left(\hat{z}\prime -{\hat{z}}^{2}\right)}}$, *α = z*_0_/*ω*_0_, and the error function Erf. Following the theory of z-scan FFS [6], the intensity profile of the scan is given by $\langle F\left(z\right)\rangle =F_{\infty }V_{\text{eff}}^{\left(1\right)}\left(R,z\right)/V_{\infty }$, where *F*_∞_ represents the intensity of an infinite sample and *V*_∞_ is the volume of the entire PSF. Evaluating 〈*F* (*z*)〉 for the modified Gaussian-Lorentzian PSF using Eq 17 describes the shape of the intensity profile,

$$\langle F\left(R,z\right)\rangle =F_{\infty }\int_{-\hat{R}}^{\hat{R}}{\left(1+{\left(\hat{z}\prime -\hat{z}\right)}^{2}\right)}^{-\eta }\;\text{Erf}\left(\phi_{1}(\hat{z})\right)d\hat{z}\prime .$$ (18)

**Fig 6:**
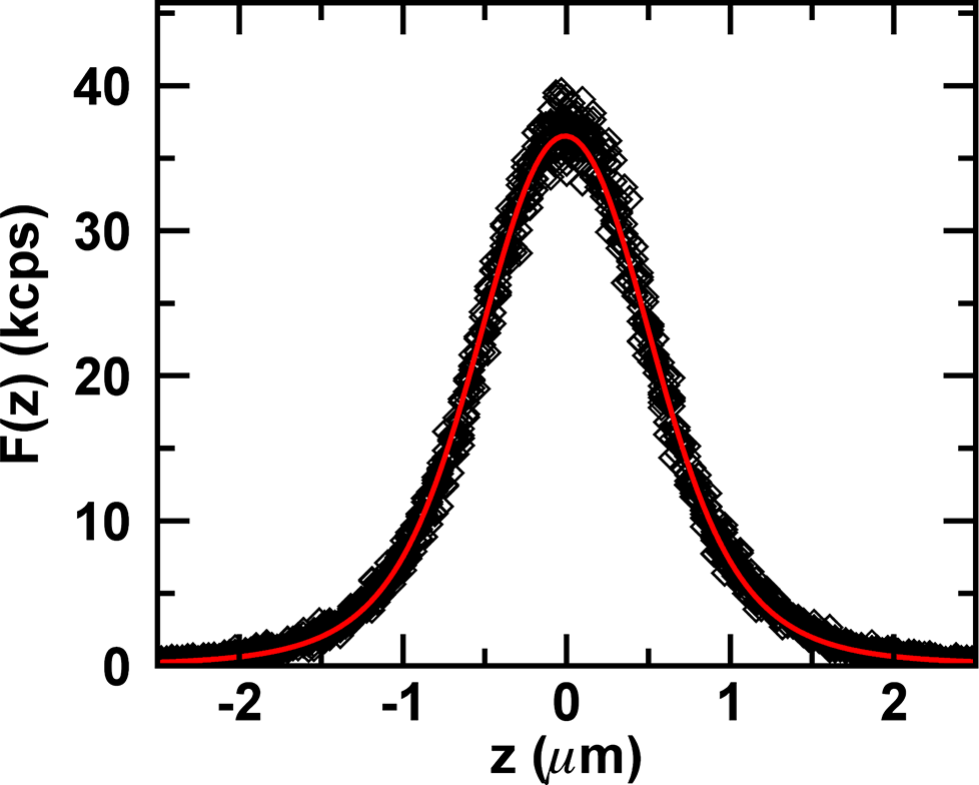
Experimental z-scan intensity profiles of EGFP from E. coli cell. Experimental z-scan intensity data (diamonds) from eight consecutive z-scans together with model function (red curve) for *F*_∞_= 72 kcps and *R* = 0.45 μm.

We performed eight consecutive z-scans through the geometric center of an E. coli cell with reduced laser power to ensure the absence of photobleaching during the scans. The intensity profiles of the consecutive scans are shown in Fig 6. Each profile was fit by Eq 18 to determine *F*_∞_ and *R*, which recovered the averaged fit parameters *F*_∞_ = 72±2 kcps and *R* = 0.45±0.01 μm. Inserting the averaged fit parameters into Eq 18 resulted in a modeled intensity profile (red solid line, Fig 6), which is in good agreement with the experimental data. We repeated this experiment on several E. coli cells (*n* = 14). The peak intensity differed for each cell, reflecting the variations in the EGFP concentration from cell to cell. However, the radius was essentially identical for all cells. The averaged radius was 0.45±0.026 μm.

Since the gamma factor is defined by $\gamma_{2}=V_{\text{eff}}^{\left(2\right)}/V_{\text{eff}}^{\left(1\right)}$ [6], its value *γ*_2_ (*z*, *R*) depends on the z position of the PSF and the radius of the E. coli bacterium. The FFS experiment was performed with the focus positioned at the geometric center of the E. coli cell (Fig 5A). Because this condition correspond to *z* = 0, the correct gamma factor that accounts for the overlap of the modified Gaussian-Lorentzian PSF with the sample is given by

$$\gamma_{2,cyl}\left(R,0\right)=\frac{V_{\text{eff}}^{\left(2\right)}\left(R,0\right)}{V_{\text{eff}}^{\left(1\right)}\left(R,0\right)}=\frac{1}{2}\frac{\int_{-\hat{R}}^{\hat{R}}{\left(1+{\hat{z}\prime }^{2}\right)}^{-1-2\eta }\;\text{Erf}\left(\phi_{2}(0)\right)d\hat{z}\prime }{\int_{-\hat{R}}^{\hat{R}}{\left(1+{\hat{z}\prime }^{2}\right)}^{-\eta }\;\text{Erf}\left(\phi_{1}(0)\right)d\hat{z}\prime }.$$ (19)

As mentioned earlier, a sample with brightness *λ* results in a Q-factor of *Q*_∞_ = *γ*_2,∞_ *λT* for an infinite sample and a Q-factor of *Q*_*cyl*_ (*R*, 0) = *γ*_2,*cyl*_ (*R*, 0) *λT* when measured at the geometric center of an E. coli cell with radius *R*. The Q-factors of both geometries are related by

$$Q_{cyl}\left(R,0\right)=Q_{\infty }\frac{\gamma_{2,cyl}\left(R,0\right)}{\gamma_{2,\infty }}.$$ (20)

**Fig 7:**
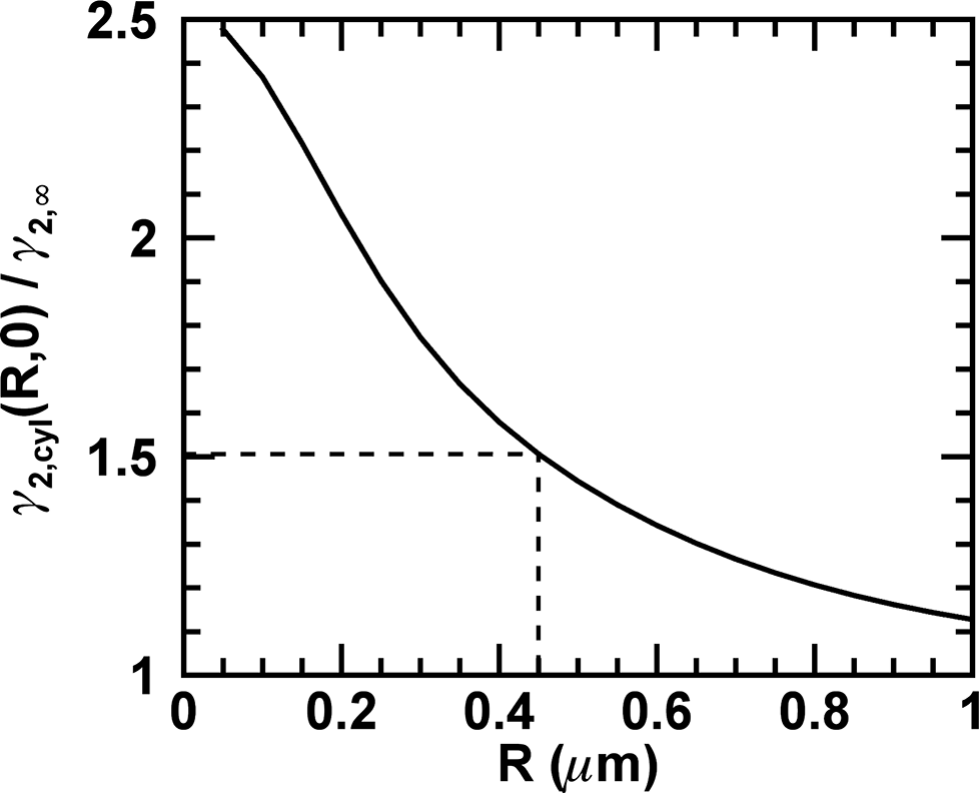
Ratio of gamma factors. The ratio *γ*_2,*cyl*_ (*R*, 0)/*γ*_2,∞_ as determined from Eq 19 is shown as a function of the radius *R*. The dashed line indicates the values for the measured E. coli cells.

The ratio *γ*_2,*cyl*_ (*R*, 0)/*γ*_2,∞_ calculated from Eq 19 is shown in Fig 7 as a function of the radius *R*. Because the radius of the E. coli bacteria was constant at 0.45 μm, the gamma ratio for E. coli is *γ*_2,*cyl*_ (0.45 μm, 0)/*γ*_2,∞_ = 1.51.

We applied Eq 20 to the calibration Q-value of EGFP (*Q*_*EGFP*,∞_ = 0.019) measured in solution to get the reference Q-factor appropriate for E. coli measurements, *Q*_*EGFP*,*cyl*_ = 0.0287. Next, we converted the Q-value (*Q*_1_ = 0.028) recovered from the fit to the MSQ-curve (Fig 3C) with Eq 4 into a normalized brightness using *Q*_*EGFP*,*cyl*_ as the reference, which resulted in *b* = 0.98. Thus, accounting for the cell geometry in MSQ analysis recovered a monomeric brightness.

**Fig 8:**
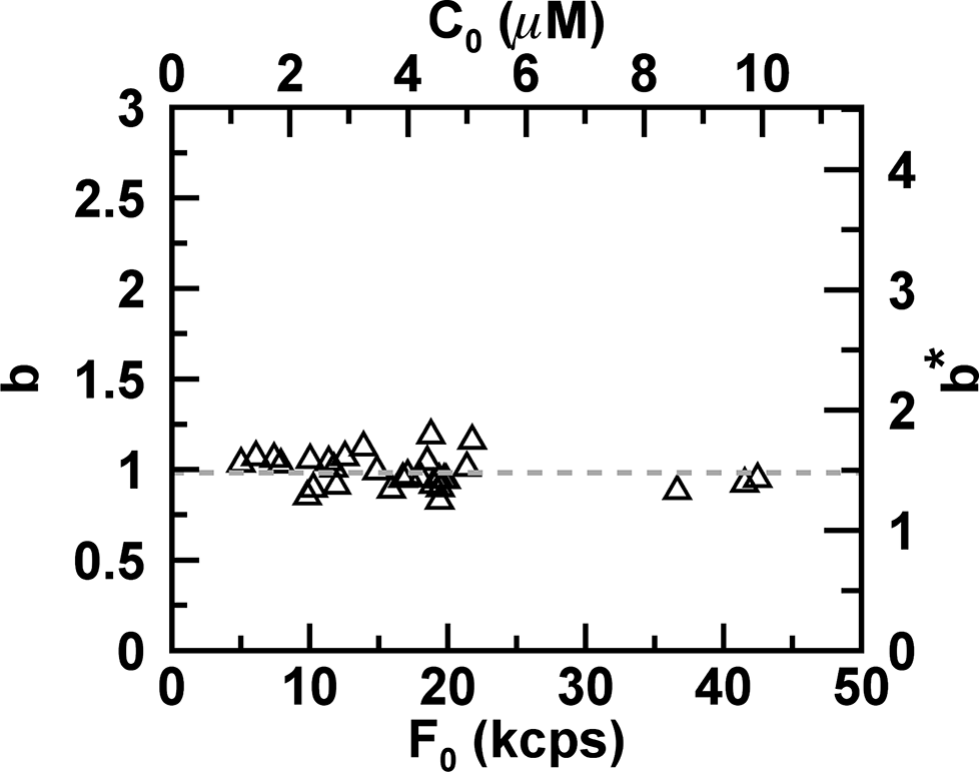
Normalized brightness of EGFP from E. coli cells. MSQ-curves were fit to Eq 14 with A(*Q*_1_, 1) = *Q*_1_ and converted into a normalized brightness by *b* = *Q*_1_/*Q*_*cyl*,∞_. The normalized brightness is independent of the initial fluorescence intensity *F*_0_. The average brightness (dashed line) is 0.98 ± 0.09. The top axis represents the initial protein concentration, while the right axis displays the biased normalized brightness *b* ^*^ = *Q*_1_/*Q*_*EGFP*,∞_, when the finite size of the bacterium is ignored.

Additional E. coli cells expressing EGFP were measured to test our analysis procedure. The FFS data taken at the geometric center were fit to Eq 14 and the normalized brightness was calculated from the recovered *Q*_1_ with the help of Eqs 4 and 20. The radius of the E. coli cell was either determined from the z-scan intensity profile or taken as 0.45 μm. We plotted the normalized brightness *b* versus the initial fluorescence intensity *F*_0_ (Fig 8). The values of *b* are close to 1 with a mean of 0.98 and a standard deviation of 0.09. This result correctly identifies the bacterially expressed EGFP as a monomeric protein. The right axis shows the biased normalized brightness *b* ^*^ = *Q*_1_/*Q*_*EGFP*,∞_ that results if the incomplete PSF overlap is not accounted for. A value of *b* ^*^ close to 1.51 would lead to the misleading conclusion that the sample is a mixture of monomers and dimers. Thus, accounting for photodepletion and geometry of the bacterium is crucial to avoid misinterpretation of FFS brightness experiments inside E.coli.

The theory developed up to this point is still incomplete. So far, we described an unbiased procedure to determine the brightness from a bacterial cell for the special case of a monomeric protein. Our model does not yet work for samples containing protein complexes, because of an additional effect of photodepletion on brightness [8]. To illustrate this issue consider a dimeric protein that contains two fluorescent labels. Photobleaching of one of the two labels results in a dimer with a reduced brightness, since only one label remains fluorescent. This process creates different brightness populations of the dimeric protein with population levels that depend on the amount of photodepletion. We recently examined this process for an *n*-meric protein with brightness *nλ* and showed that photodepletion leads to a time-dependence of the first two cumulants [8],

$$\begin{array}{l} \kappa_{\left[1\right]}\left(t\right)=\sum_{s=0}^{n}\lambda \left(n-s\right)Tp_{n-s}N_{0},\\ \kappa_{\left[2\right]}\left(t\right)=\sum_{s=0}^{n}\gamma_{2}\lambda^{2}{\left(n-s\right)}^{2}\;T^{2}p_{n-s}N_{0}, \end{array}$$ (21)

with *λ* representing the brightness of a single label, *N*_0_ being the initial number of n-mers in the observation volume and $p_{n-s}=\left(\begin{array}{c} n\\ s \end{array}\right)p^{s}{\left(1-p\right)}^{n-s}$, where $p=1-e^{-k_{D}t}$ is the probability that a fluorescent label is photobleached at time *t*. Following the same steps applied to the cumulants of a monomeric protein (Eq 11) to the cumulants of an n-mer (Eq 21) produced an MSQ function MSQ_*PD*_ (*T*_*S*_) accounting for photodepletion that is identical to Eq 12 (see section C of S1 File). Next, we included the effect of estimator bias (see section D of S1 File), which produced an MSQ function that is identical to Eq 14. The only difference to the monomeric case is found in the function A(*Q*_1_, *n*), which for a protein with stoichiometry *n* is given by,

$$\text{A}\left(Q_{1},n\right)=Q_{1}\left[1+\left(n-1\right)\frac{2-\Delta f_{D}}{2}\times \frac{1-{\left(1-\Delta f_{D}\right)}^{M}}{M\Delta f_{D}}\right].$$ (22)

**Fig 9:**
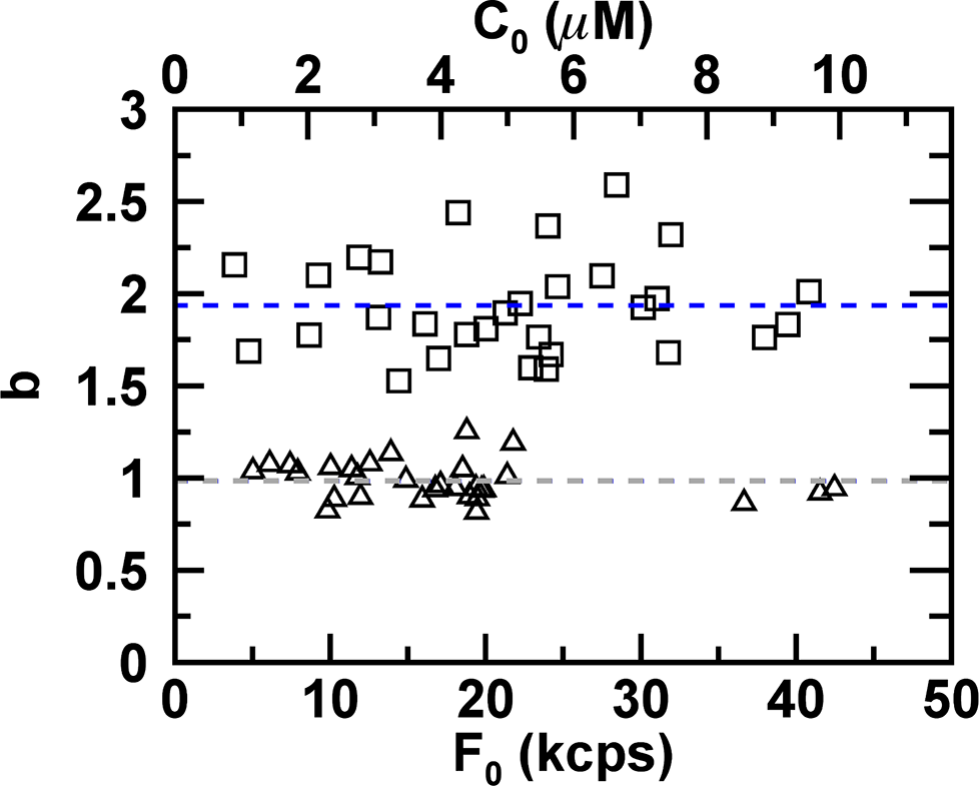
Measured stoichiometry of proteins in E. coli cells. MSQ-curves were fit to Eqs 14 and 22 to determine the average stoichiometry *n* for EGFP (triangles) and NTF2-EGFP (squares) as a function of the initial fluorescence intensity *F*_0_. The average stoichiometry of EGFP (gray dashed line) is 0.98 ± 0.10. The average stoichiometry of NTF2-EGFP (blue dashed line) is 1.94 ± 0.27. The top axis displays the initial protein concentration.

Now that we have a complete theory, we decided on the following strategy to analyze the MSQ-curve from an E. coli sample with unknown stoichiometry *n*. The experimental MSQ-curve is fit to Eqs 14 and 22 with *n*, *k*_*D*_, and *τ*_*D*_ as the only fit parameters. *F*_0_ is determined from a fit of the intensity decay curve, while *N* = *T*_*S*_/*T*, *M* = *T*_*DAQ*_/*T*_*S*_, and Δ*f*_*D*_ = 1−exp(−*k*_*D*_ *T*_*S*_) are functions of *T*_*S*_. The monomeric Q-factor *Q*_1_ of the function A is needed as a calibration factor and set equal to *Q*_*EGFP*,*cyl*_ to account for the geometry of the bacterium. Because the normalized brightness *b* and the stoichiometry *n* are numerically identical, *b* = *n*, we use both parameters interchangeably and at times refer to *n* as the normalized brightness. As a first test of this procedure we reanalyzed the FFS data from E. coli expressing EGFP with the new fit strategy to recover the stoichiometry of the sample. The analysis returned a normalized brightness *n* of ~1 for all samples (mean of 0.98 ± 0.10) as expected for a monomeric protein (Fig 9). The fit parameter *k*_*D*_ varied slightly from cell to cell (mean 0.022 s^-1^ and standard deviation 0.0073 s^-1^), because of volume variations caused by different lengths of the E. coli cells. The diffusion time *τ*_*D*_ was approximately the same with a mean of 2.5 ± 0.9 ms.

We turned to the MSQ-curve taken in E. coli expressing NTF2-EGFP (Fig 3D). A fit (solid line, Fig 3D) to Eqs 14 and 22 with *Q*_1_ = *Q*_*EGFP*,*cyl*_ resulted in a normalized brightness of *n* = 2.1, a diffusion time of 10 ms, and a depletion rate coefficient of 0.053 s^-1^. The initial intensity *F*_0_ was determined from a fit of the intensity decay curve (S3 Fig). The normalized brightness indicates a dimeric protein complex, which is consistent with the observation of dimeric NTF2-EGFP in U2OS cells and in solution [6,20]. We applied the same analysis to additional FFS data from E. coli cells expressing mammalian NTF2-EGFP. The fitted normalized brightness was ~2 in all cases (Fig 9), indicating that NTF2-EGFP exists as a dimer in the E. coli cytosol. The mean of the normalized brightness was 1.94 ± 0.27 and the fitted diffusion time *τ*_*D*_ had a mean of 9.5 ± 3.4 ms.

Finally, the theory developed here also allowed us to convert the initial fluorescence intensity *F*_0_ into a concentration. The value of *F*_0_ is given by *F*_0_ = *λ*_*EGFP*_ *N*_0_ with *N*_0_ representing the initial number of the EGFP-labeled proteins in the PSF volume. The molar concentration is determined by dividing *N*_0_ by Avogadro’s number *N*_*A*_ and the effective PSF volume $V_{\text{eff}}^{\left(1\right)}\left(R,0\right)$ of the mGL-PSF focused at the geometric center of an E. coli of radius *R*, $c=N_{0}/\left(V_{\text{eff}}^{\left(1\right)}\left(R,0\right)\cdot N_{A}\right)$. The top axis of Figs 8 and 9 depicts the concentration of EGFP and NTF2-EGFP of the E. coli measurements, respectively.

## Discussion

This work provides a detailed account of the development of quantitative brightness analysis of FFS data in prokaryotic cells with E. coli chosen as our model system. The function MSQ(*T*_*S*_) is central to our analysis strategy, and is experimentally determined from FFS data by Eqs 6 and 8. The final formulation of MSQ theory (Eqs 22 and 14) takes photobleaching, which depletes the fluorophore population and reduces the brightness of protein complexes, and estimator bias into account. The MSQ method is a significant advancement over SBA analysis. In essence, SBA analysis can only be applied, if the MSQ-curve has a plateau. Thus, simple inspection of the MSQ-curves of Fig 3B and 3C demonstrates that the SBA model is sufficient for the yeast measurement, but fails in case of the E. coli data. In contrast, the MSQ model was successfully applied to FFS data obtained from mammalian, yeast, and E. coli cells.

MSQ is directly determined from the experimental photon count data without the need for any model. Thus, connecting MSQ with the brightness, which depends on the PSF and its overlap with the sample, requires additional information. Two external factors *F*_0_ and *Q*_*EGFP*,*cyl*_ are needed for fitting the MSQ curve. *F*_0_ represents the initial fluorescence intensity and is determined from a fit of the intensity decay curve. *Q*_*EGFP*,*cyl*_ is the reference Q-factor of the monomer that accounts for the geometry of the E. coli cell. Its calculation by Eq 20 requires *γ*_2,*cyl*_ (*R*, 0)/*γ*_2,∞_, which accounts for the overlap between the geometry of the sample and the PSF. We extended the theory of z-scan FFS to E. coli, determined the radius *R* from the z-scan intensity profile and calculated *γ*_2,*cyl*_ (*R*, 0)/*γ*_2,∞_. The ratio is ~1.5, which reflects a significant correction of brightness due to the geometry.

Effects associated with the geometric size of prokaryotic cells have not been accounted for in previous fluorescence fluctuation studies [21,22], except for one study where a Monte-Carlo simulation was used to estimate a correction factor accounting for the bacterial geometry assuming a 3D-Gaussian PSF [23]. However, commonly used model functions, such as the 3D-Gaussian model, do not accurately reflect the actual PSF shape [6]. Thus, quantitative studies should use an experimentally characterized PSF instead of a general model function, because the numerical values of the effective volume and gamma factor depend on the form of the PSF. This study used the modified Gaussian-Lorentzian function with calibrated parameters (Eq 1), which we have found to be a sufficiently precise model for two-photon microscopy [6,7].

In the derivation of the general MSQ model we assumed an irreversible photobleaching reaction of the label from a single bright to a non-fluorescent state. We earlier demonstrated with experiments both in mammalian and in yeast cells that the brightness behavior of EGFP was well approximated by this simple model provided the intensity loss by photodepletion was less than 60% [8]. Because the intensity reduction of our E. coli experiments never exceeded ~50%, the simple photobleaching model is sufficient. However, for experiments using a different fluorescent protein as label it is important to perform control experiments to establish the validity of the photobleaching model employed here.

The analysis of MSQ data of E. coli cells by Eqs 14 and 22 included three fitting parameters, *n*, *τ*_*D*_, and *k*_*D*_. Because the depletion rate coefficient *k*_*D*_ can also be determined from the fit of the intensity decay curve *F*(*t*), *k*_*D*_ can be changed to a fixed instead of a free parameter. We chose to keep it as a free parameter in the MSQ analysis and found that the recovered values of *k*_*D*_ by MSQ and from the intensity decay agreed within a few percent.

The main goal of this study was the determination of the normalized brightness or stoichiometry of soluble proteins in E. coli. The analysis accurately identified EGFP as a monomer and NTF2-EGFP as a dimer (Fig 9). The mean of the normalized brightness values deviated less than 5% from the ideal monomer and dimer brightness. Remarkably, the uncertainty in the normalized brightness of a single measurement was ~10% for EGFP and ~14% for EGFP-NTF2, which is similar to the brightness uncertainty achieved in U2OS cells [15,16]. Thus, MSQ analysis of fluctuation data from a bacterium achieved an accuracy that matched standard FFS experiments in mammalian cells. This result speaks to the robustness of the MSQ approach. We choose NTF2 for this study, because it forms a very tight dimer and is not found in prokaryotes. The absence of unlabeled endogenous NTF2 ensures that all dimers formed contain two labels, as confirmed by our analysis, which represents the first quantitative brightness analysis of protein interactions in E. coli cells.

We were able to calculate the absolute concentration of a labeled protein in E. coli, which is a byproduct of MSQ analysis. Measuring concentrations inside bacterial cells is of sufficient interest that many studies have been devoted to this topic. By comparing the fluorescence intensity of bacteria with a reference sample of known concentration and applying correction factors absolute concentrations can be estimated (reviewed in [24]). A powerful method for measuring absolute concentrations in bacterial cells is based on single molecule counting [25], but this approach is technically challenging and works best at low protein concentrations. A different approach to measure concentrations uses intensity fluctuations between daughter cells after bacterial cell division [26,27]. Concentrations determined from intensity fluctuation of imaged bacteria have also been reported [23,28]. Because the amplitude of fluctuations is directly related to concentration, fluctuation methods are a powerful approach to measure absolute concentrations without relying on a concentration reference. However, these methods are often intricate and care needs to be exercised to minimize systematic errors [29]. The MSQ method has been carefully characterized and accounts for sample geometry, PSF shape, photobleaching, and statistical bias, which should eliminate many sources of systematic error. Thus, MSQ may offer an attractive approach for measuring bacterial protein concentrations.

MSQ analysis also recovers the diffusion time *τ*_*D*_ through the dependence of the MSQ on the binning function *B*_2_(*t*, *τ*_*D*_). The standard method of measuring the diffusion time in FFS is the autocorrelation function. Because the binning function *B*_2_ is mathematically related to the autocorrelation function [12,14], it also can recover the diffusion time. We found in U2OS cells, where photodepletion is negligible and only estimator bias contributes to the shape of the MSQ curve (Fig 3A), that MSQ and autocorrelation analysis recover diffusion times that agree within ~10%. Extending autocorrelation analysis from eukaryotic cells to bacterial samples faces challenges. Photodepletion, brightness reduction of protein complexes due to photobleaching, and the presence of potential estimator bias can affect the fluctuation amplitude of each diffusing species. The derivation of MSQ theory provides a rigorous framework for incorporating these effects into autocorrelation analysis. Of course, the partial overlap of the sample with the PSF affects the amplitude as well. An additional complication is the effect of diffusion in a confined space on the shape of the autocorrelation function [30]. Because this effect appears to be sufficiently small in E. coli cells, determination of diffusion times should generally be possible [21,22]. Thus, we expect that the finite size of E. coli has no significant effect on the diffusion-time dependent shape of the MSQ curve. In fact, we can justify this statement with a simple estimate. Fig 4 shows the influence of diffusion on the MSQ amplitude. Given the experimental uncertainty, it seems reasonable to disregard effects that introduce less than ~20% bias. This implies that diffusion significantly influences the MSQ curve only for segment times *T*_*S*_ ≤ 20*τ*_*D*_. The maximum time *T*_*S*_ ∼ 20*τ*_*D*_ leads to a root-mean square displacement Δ*x*_*rms*_ of $\sqrt{2DT_{S}}$ = $\frac{1}{2}w_{0}\sqrt{T_{S}/\tau_{D}}$ ~ 1 μm, which is approximately the shortest dimension of the E. coli cell. Thus, diffusion for *T*_*S*_ ≤ 20*τ*_*D*_ is essentially not confined, which justifies our diffusion analysis of the MSQ curve.

MSQ analysis of EGFP in E. coli recovered an average diffusion time of 2.5 ms with a standard deviation of ~35%. Converting the diffusion time into a diffusion coefficient, $\tau_{D}=\omega_{0}^{2}/8D$, results in a value of $9.2_{-2.5}^{+5.2}$ μm^2^/s. The diffusion coefficient of GFP in E. coli has been measured in many studies with techniques that include FRAP, FCS, and single molecule tracking [31–33]. The majority of values reported by these studies range from 6 to 14 μm^2^/s [33], which is consistent with our result. Finally, the diffusion time is not affected by photodepletion, because the timescales are vastly different. Diffusion times occur on the millisecond timescales, while the characteristic photodepletion time is on the order of tens of seconds. Thus, the probability of a given fluorophore to undergo photobleaching while passing through the PSF is vanishingly low. This point has been discussed in more detail recently [8].

The MSQ method offers a fairly straightforward and simple analysis approach. The algorithm for calculating the MSQ-curve is easy to implement and consists of data rebinning followed by the calculation of MSQ values from the average and variance of the rebinned data. In addition, the FFS data taken inside the small sample compartment contain all relevant information, except for the influence of geometry on FFS parameters, which is established by taking a separate z-scan fluorescence intensity profile. The initial intensity *F*_0_ and the MSQ-curve determined from the FFS data identify the concentration, brightness, diffusion time, and depletion rate coefficient with the help of Eq 14. We successfully demonstrated combined MSQ and z-scan analysis on proteins that are soluble and uniformly distributed inside E. coli. Extending MSQ analysis to a non-uniform protein distribution, such as generated by a protein bound to the cell membrane of E. coli, would require additional development work and is beyond the scope of this study. However, a recent paper discusses brightness experiments of non-uniformly distributed proteins [7], which might serve as a suitable starting point for the development of a generalized MSQ theory.

Combining laser scanning with fluorescence fluctuation measurements is a very popular approach [5,28,34,35]. One of the reported advantages of scanning is the reduction of photobleaching effects on fluctuation experiments [36]. Because the probability of photobleaching increases with exposure time, scanning the beam causes a reduction in the amount of photobleaching at any one particular volume. However, this advantage becomes marginal when the sample size is similar to the size of the laser beam as is the case for bacterial cells. Scanning also does not reduce the depletion of fluorophores compared to stationary measurements. The amount of fluorophores depleted from a sample depends directly on the integrated laser power the sample was exposed to irrespective whether scanning occurred or not. However, a significant advantage of scanning over stationary measurements is the possibility to probe all bacteria in the field of view simultaneously, while our approach requires manual repositioning and aligning of the cell with respect to the beam, which is a time-consuming process. A recent study used this advantage of scanning to determine protein concentration in Bacillus subtilis cells with N&B analysis [23,28]. Because the N&B algorithm is similar to calculating the Q-factor, it should be possible to adapt MSQ theory to scanning applications that account for photodepletion and estimator bias, and thereby reducing systematic errors in the data analysis.

In summary, the MSQ method enables quantitative brightness analysis of soluble proteins in samples ranging from mammalian to bacterial cells. Geometric overlap between the bacterial cell and the PSF, which was characterized by z-scan FFS, has to be considered to correctly connect the Q-value with the stoichiometry of a protein complex. MSQ analysis was used to successfully identify monomers, as well as dimers, in E. coli cells. In addition, MSQ provides the diffusion times of the labeled protein. The results of this work demonstrate that quantitative FFS analysis of protein complexes and their concentrations in femtoliter-sized compartments is feasible. We expect that the MSQ approach will prove useful as a robust analysis method for FFS studies of bacterial samples. The concepts of MSQ theory might also provide a useful starting point for future FFS studies of small organelles in mammalian cells, such as the endoplasmic reticulum or the nucleolus.

## Supporting Information

S1 Fig Fluorescence intensity trace *F*(t) of EGFP in U2OS cell. (PDF)

S2 Fig Fluorescence intensity trace *F*(t) of EGFP in yeast cell. (PDF)

S3 Fig Fluorescence intensity trace *F*(t) of NTF2-EGFP in E. coli cell. (PDF)

S1 File Derivation of MSQ theory. (PDF)
