# Supplementary material for: Quantitative Brightness Analysis of Fluorescence Intensity Fluctuations in E. Coli
Source: PLoS One. 2015 Jun 22;10(6):e0130063. doi: 10.1371/journal.pone.0130063 (PMC4476568; doi:10.1371/journal.pone.0130063)
Supplement: S1 Fig — (PDF) [file pone.0130063.s001.pdf]

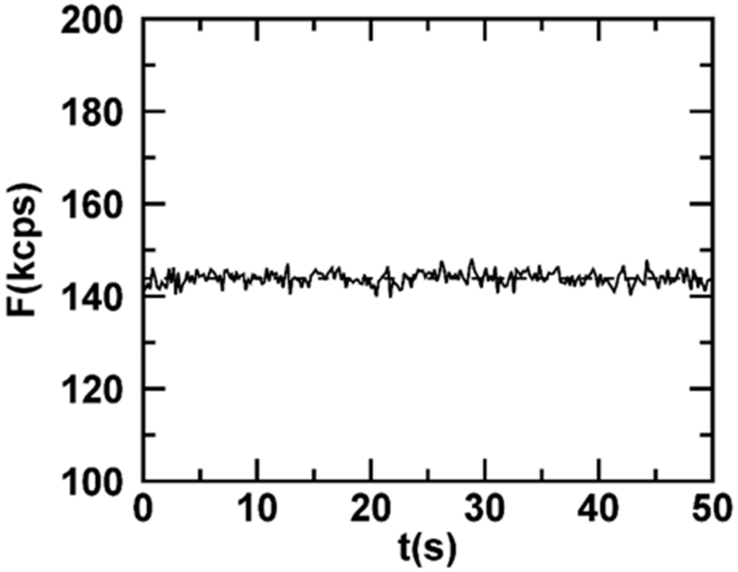

S1 Fig. Fluorescence intensity trace *F*(t) of EGFP in U2OS cell. The fluorescence intensity of EGFP measured inside the nucleus is stationary with an average value of 144 kcps.
