# Supplementary material for: Quantitative Brightness Analysis of Fluorescence Intensity Fluctuations in E. Coli
Source: PLoS One. 2015 Jun 22;10(6):e0130063. doi: 10.1371/journal.pone.0130063 (PMC4476568; doi:10.1371/journal.pone.0130063)
Supplement: S3 Fig — (PDF) [file pone.0130063.s003.pdf]

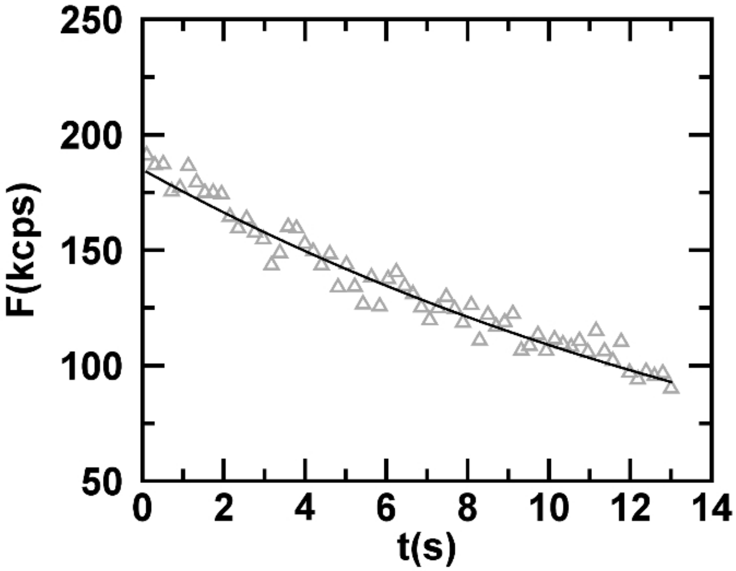

S3 Fig. Fluorescence intensity trace *F*(t) of NTF2-EGFP in E. coli cell. The experimental intensity data are described by an exponential decay (solid line) with $F_0 = 185$ kcps and a rate coefficient $k_D = 0.053\ s^{-1}$.
