# Supplementary material for: Quantitative Brightness Analysis of Fluorescence Intensity Fluctuations in E. Coli
Source: PLoS One. 2015 Jun 22;10(6):e0130063. doi: 10.1371/journal.pone.0130063 (PMC4476568; doi:10.1371/journal.pone.0130063)
Supplement: S1 File — (PDF) [file pone.0130063.s004.pdf]

S1 File. Derivation of MSQ theory.

A. Q-factor estimator for stationary signals

We rewrite the estimators of the first two photon count moments of the m-th segment, $\widehat{k_m} = N^{-1}\sum_{i=1}^{N} k_{m,i}$ and $\widehat{k_m^2} = N^{-1}\sum_{i=1}^{N} k_{m,i}^2$ by substituting $k_{m,i} = \langle k_m \rangle + \delta k_{m,i}$ with $\delta k_{m,i}$ being the instantaneous fluctuation around the mean $\langle k_m \rangle$ and inserting it into the definition of the Q-estimator of the m-th segment given by Eq. 6 of the manuscript. Because the estimators $\widehat{k_m}$ and $\widehat{k_m^2}$ are unbiased by the mean ergodic theorem [1], their expectation values are equal to the population moments of the photon counts, $E\widehat{k_m} = \langle k_m \rangle$ and $E\widehat{k_m^2} = \langle k_m^2 \rangle$. The estimator $\widehat{k_m}$ in the denominator can be expressed as $\widehat{k_m} = \langle k_m \rangle + \Delta$ with $\Delta = N^{-1}\sum_{i=1}^{N} \delta k_{m,i}$. As N increases, Δ must vanish to satisfy the mean ergodicity theorem, which implies $\Delta / \langle k_m \rangle \ll 1$. Taking the Taylor expansion of the denominator up to the second order of $\Delta / \langle k_m \rangle$ leads to

$$\widehat{Q_m}(T_S) = \frac{N^{-1}\sum_{i=1}^{N} \delta k_{m,i}^{\;2} - \langle k_m \rangle}{\langle k_m \rangle} - \frac{N^{-2}\sum_{i,j=1}^{N} \delta k_{m,i}\delta k_{m.j}}{\langle k_m \rangle} + O\left(\left(\frac{\Delta}{\langle k_m \rangle} \right)^3 \right). \tag{SI.1}$$

The expectation value of this estimator is its ensemble average or population mean given by

$$E\widehat{Q_m}(T_S) = \left\langle \widehat{Q_m}(T_S) \right\rangle = Q_m - \frac{N^{-2}\sum_{i,j=1}^{N} \left\langle \delta k_{m,i}\delta k_{m.j} \right\rangle}{\langle k_m \rangle} \tag{SI.2}$$

where we used $\langle \delta k_{m,i}^2 \rangle = \langle \Delta k_m^2 \rangle$ and introduced the Q-factor of the m-th segment by applying the same definition as used in traditional FFS theory (see Eq. 3 of the manuscript),

$$Q_m = \frac{\kappa_{[2],m}}{\kappa_{[1],m}} = \frac{\left\langle \Delta k_m^2 \right\rangle - \left\langle k_m \right\rangle}{\left\langle k_m \right\rangle} = Q = \gamma_2 \lambda T \ . \qquad \text{(SI.2)}$$

Because the signal is stationary, Q_m has to have the same value Q for all segments.

We next rewrite the summation in Eq. (SI.2) as a sum of variances and covariances

$$\sum_{i,j=1}^{N} \left\langle \delta k_{m,i} \delta k_{m,j} \right\rangle = \sum_{i=1}^{N} \left\langle \delta k_{m.i}{}^2 \right\rangle + 2\sum_{i=1<j}^{N} \left\langle \delta k_{m.i} \delta k_{m.j} \right\rangle . \qquad \text{(SI.3)}$$

Each of the two sums is expressed as factorial cumulants of the photon counts as detailed in [2],

$$\begin{aligned} &\sum_{i=1}^{N} \left\langle \delta k_{m,i}{}^2 \right\rangle = N\kappa_{[2],m}\left(T\right) + N\kappa_{[1],m}\left(T\right) \\ &2\sum_{i=1<j}^{N} \left\langle \delta k_{m.i} \delta k_{m,j} \right\rangle = \kappa_{[2],m}\left(T_S\right) - N\kappa_{[2],m}\left(T\right) \end{aligned} . \qquad \text{(SI.4)}$$

The first two factorial cumulant of the m-th segments are given by $\kappa_{[1].m}\left(T\right) = \lambda T N_0$ and $\kappa_{[2],m}\left(t\right) = \gamma_2 \lambda^2 N_0 B_2\left(t, \tau_D\right)$. B_2 is called the second-order binning function [2,3]. The first moment $\left\langle k_m \right\rangle$ is equal to the first factorial cumulant $\kappa_{[1],m}$ [2,3]. We insert the above relations into Eq, (SI.2) to express the expectation value of the Q-estimator

$$E\widehat{Q_m}\left(T_S\right) = \left\langle \widehat{Q_m}\left(T_S\right) \right\rangle = Q_m - \frac{1}{N} - Q_m \frac{B_2\left(T_S, \tau_D\right)}{T_S^2} , \qquad \text{(SI.5)}$$

where we used Eq. (SI.2) and the absence of undersampling as assumed throughout the manuscript,

$$Q_m = \frac{\kappa_{[2],m}\left(T\right)}{\kappa_{[1],m}\left(T\right)} = \gamma_2 \lambda \frac{B_2\left(T, \tau_D\right)}{T} = \gamma_2 \lambda T \ . \qquad \text{(SI.5)}$$

The binning function reduces to $B_2(T,\tau_D)=T^2$ in the absence of undersampling [2]. Finally, we take the average of Eq. (SI.5) over all segments to derive the expectation value of the MSQ-function due to estimator bias $\mathrm{MSQ}_{EB}(T_S)=\langle \mathrm{MSQ}(T_S)\rangle = M^{-1}\sum_{m=1}^{M}\langle \widehat{Q_m}(T_S)\rangle$. Since Q_m has the same value Q for all segments for a stationary signal, Eq. (SI.5) is independent of the segment number, which results in

$$\mathrm{MSQ}_{EB}(T_S)=Q-\frac{1}{N}-Q\frac{B_2(T_S,\tau_D)}{T_S^2}. \qquad \text{(SI.5)}$$

B. Time-averaged Q-estimator for a non-stationary signal

We consider the data segment defined by the time interval $\left[(m-1)T_S, mT_S\right]$. For simplicity, we assume a long enough segment so that estimator bias is negligible. Estimators for the mean and variance of the photon counts over a segment are defined by

$$\begin{aligned}\widehat{k_m} &\equiv \frac{1}{N}\sum_{i=1}^{N}k_{m,i} = \frac{1}{T_S}\int_{(m-1)T_S}^{mT_S} k_m(t')\,dt' \\ \widehat{\Delta k_m^2} &\equiv \frac{1}{N}\sum_{i=1}^{N}\left(k_{m,i}-\overline{k_m}\right)^2 = \frac{1}{T_S}\int_{(m-1)T_S}^{mT_S}\left(k_m(t')-\overline{k_m}\right)^2 dt'\end{aligned}. \qquad \text{(SI.5)}$$

The sum is converted into an integration since the sampling time T is much smaller than the segment time T_S. A bar over a variable defines the time-average over the segment period as defined in Eq. 9 of the manuscript. Thus, $\overline{k_m}$ denotes the time-average of the photon counts (see Eq. (SI.5)). The variance is estimated by subtracting the time-averaged mean from the instantaneous photon count $k_m(t)$. The expectation values of the above estimators are

$$E\hat{k}_m = \frac{1}{T_S}\int_{(m-1)T_S}^{mT_S} \langle k_m(t') \rangle dt' = \overline{k_m}$$
$$E\widehat{\Delta k^2}_m = \frac{1}{T_S}\int_{(m-1)T_S}^{mT_S} \left\langle \left(k_m(t') - \overline{k_m}\right)^2 \right\rangle dt' = \overline{k_m^2} - \overline{k_m}^2 . \qquad \text{(SI.5)}$$

Next, we express the time-average of the first two photon count moments in terms of time-averaged factorial cumulants,

$$\overline{k_m} = \overline{\kappa_{[1],m}}$$
$$\overline{k_m^2} = \overline{\kappa_{[2],m}} + \overline{\kappa_{[1],m}^2} + \overline{\kappa_{[1],m}} + \overline{\kappa_{[1],m}}^2 . \qquad \text{(SI.5)}$$

The above relations are based on known relations between raw moments and factorial cumulants [3]. Specifically, the mean of the photon counts equals the first factorial cumulant of the photon counts, $\langle k(t') \rangle = \kappa_{[1]}(t')$, while the second moment is given by $\langle k^2 \rangle = \kappa_{[2]} + \kappa_{[1]}^2 + \kappa_{[1]}$. Applying Eq. 9 of the manuscript to these relations results in Eq. (SI.5). Finally, evaluating the expectation value of the Q-estimator (Eq. 6) with the help of Eqs. (SI.5) and (SI.5) leads to

$$E\widehat{Q_m} = \frac{E\left(\widehat{\Delta k_m^2} - \widehat{k_m}\right)}{E\widehat{k_m}} = \frac{\overline{\kappa_{[2],m}} + \overline{\kappa_{[1],m}^2} - \overline{\kappa_{[1],m}}^2}{\overline{\kappa_{[1],m}}} . \qquad \text{(SI.5)}$$

C. MSQ function in the presence of photodepletion

Before deriving the MSQ function we must evaluate Eq. (SI.5) for a monomeric or n-meric protein sample. Let us assume a fluorescently-labeled protein F that associates to form an n-mer F_n with a brightness λn, where λ is the brightness of the monomer. We postulate that a chromophore in the fluorescent state F converts irreversibly and independently to a non-fluorescent dark state D as a result of photobleaching. Thus, photobleaching of exactly one

chromophore leads to the state $F_{n-1}D_1$ with brightness $\lambda(n-1)$. The probability for a fluorophore to be photobelached is given by $p = 1-\exp(-k_D t)$. The n-mer's brightness state F_n changes into the state $F_{n-s}\,D_s$ of brightness $\lambda(n-s)$ with the probability $p_{n-s} = \binom{n}{s} p^{\,s}(1-p)^{n-s}$. The initial state at $t=0$ is the n-mer F_n with the number of molecules equal to N_0. The number of molecules of each state at time *t* is given by the number $N_0 p_{n-s}$. The factorial cumulants for an n-meric protein in the presence of photodepletion (Eq. 21 of the manuscript) were previously derived assuming the absence of undersampling [4]. These two cumulants simplify to

$$\begin{aligned} \kappa_{[1]}(t) &= n\lambda T N_0 e^{-k_D t}, \\ \kappa_{[2]}(t) &= \gamma_2 \lambda^2 T^2 N_0 \left\{ \left(n^2 - n\right) e^{-2k_D t} + n e^{-k_D t} \right\} \end{aligned} \tag{SI.6}$$

Next we calculated time-integrated cumulant values of Eq. (SI.6) for the m-th segment as defined by Eq. 9 of the manuscript,

$$\begin{aligned} \overline{\kappa_{[1]m}} &= n\lambda T N_0 e^{-k_D(m-1)T_S} \left(1-e^{-k_D T_S}\right) \Big/ k_D T_S \\ \overline{\kappa^2_{[1]m}} &= \left(n\lambda T N_0\right)^2 e^{-2k_D(m-1)T_S} \left(1-e^{-2k_D T_S}\right) \Big/ 2k_D T_S \\ \overline{\kappa_{[2]m}} &= \gamma_2 \lambda^2 T^2 N_0 \left\{ \begin{aligned} & n e^{-k_D(m-1)T_S} \left(1-e^{-k_D T_S}\right) \Big/ k_D T_S \\ & + \left(n^2 - n\right) e^{-2k_D(m-1)T_S} \left(1-e^{-2k_D T_S}\right) \Big/ 2k_D T_S \end{aligned} \right\} \end{aligned}, \tag{SI.7}$$

where $\overline{\kappa_{[1]m}}$ and $\overline{\kappa^2_{[1]m}}$ are affected by the overall intensity drop due to photodepletion, while $\overline{\kappa_{[2]m}}$ is sensitive to the variation in brightness states caused by photodepletion. We inserted the expressions of Eq. (SI.7) into Eq. 10 of the manuscript to evaluate the expectation value of the Q-estimator of the m-th segment,

$$\widehat{EQ_m} = Q_1 \left\{ 1 + (n-1) \frac{2-\Delta f_D}{2} e^{-k_D(m-1)T_S} \right\} + F_0 T e^{-k_D(m-1)T_S} \left(\frac{2-\Delta f_D}{2} + \frac{\Delta f_D}{\ln(1-\Delta f_D)} \right), \quad \text{(SI.8)}$$

where we used $Q_1 = \gamma_2 \lambda T$ for the monomeric Q-factor and the photodepletion fraction $\Delta f_D = 1 - e^{-k_D T_S}$. For the special case n = 1 and m = 1, Eq. (SI.8) reduces to a previously derived equation [4]. From here on we have to distinguish carefully between the Q-factor Q_1 of a monomer and the Q-factor $Q = nQ_1$ of an n-mer.

We determine a function describing the MSQ-curve in the presence of photodepletion by averaging Eq. (SI.8) over all segments, $\mathrm{MSQ}_{PD}(T_S) = M^{-1} \sum_{m=1}^{M} \widehat{EQ_m}(T_S)$. By using the geometric sum $\sum_{m=1}^{M} e^{-k_D(m-1)T_S} = \left(1-(1-\Delta f_D)^M\right) \Delta f_D^{-1}$, we derive

$$\mathrm{MSQ}_{PD}(T_S) = \mathrm{A}(Q_1, n) + F_0 T \frac{1-(1-\Delta f_D)^M}{M \Delta f_D} \left(\frac{2-\Delta f_D}{2} + \frac{\Delta f_D}{\ln(1-\Delta f_D)} \right), \quad \text{(SI.9)}$$

which corresponds to Eq. 12 of the manuscript. The term $\mathrm{A}(Q_1, n)$ is defined by

$$\mathrm{A}(Q_1, n) = Q_1 \left\{ 1 + (n-1) \frac{2-\Delta f_D}{2} \times \frac{1-(1-\Delta f_D)^M}{M \Delta f_D} \right\}, \quad \text{(SI.10)}$$

which reduces to Q_1 for the case of a monomeric protein (n = 1).

D. MSQ function in the presence of photodepletion and estimator bias

To combine estimator and photodepletion bias we start with the Q-estimator for the m-th segment given by Eq. 6 of the main text as $\widehat{Q_m} = \left(\widehat{\Delta k_m^2} - \widehat{k_m}\right) \Big/ \widehat{k_m}$ together with $\widehat{k_m}$ and $\widehat{\Delta k_m^2}$ defined by Eq. (SI.5). The estimator $\widehat{k_m}$ is rewritten as $\widehat{k_m} = \overline{k_m} + \sum_{i=1}^{N} \Delta k_{m,i}$ with $\overline{k_m}$ representing the expectation value of the estimator and $\Delta k_{m,i} = k_{m,i} - \overline{k_m}$. By following the steps outlined in section A an equation for the expectation value of Q-estimator is found,

$$E\widehat{Q_m} = \frac{\overline{\Delta k_m^2} - \overline{k_m}}{\overline{k_m}} - \frac{N^{-2} \sum_{i,j=1}^{N} \left\langle \Delta k_{m,i} \Delta k_{m,j} \right\rangle}{\overline{k_m}}, \tag{SI.11}$$

where the second term represents the estimator bias. This equation is equivalent to Eq. (SI.2), except that it also includes photodepletion. The sum of the second term can be expressed as

$$\sum_{i,j=1}^{N} \left\langle \Delta k_{m,i} \Delta k_{m,j} \right\rangle = \sum_{i=1}^{N} \left\langle \Delta k_{m.i}{}^{2} \right\rangle + 2 \sum_{i=1<j}^{N} \left\langle \Delta k_{m.i} \Delta k_{m.j} \right\rangle. \tag{SI.12}$$

Following the procedure used for the derivation of Eq. (SI.4), we can rewrite both sums as

$$\begin{aligned} &\sum_{i=1}^{N} \left\langle \Delta k_{m.i}{}^{2} \right\rangle = N\overline{\kappa_{[2],m}} - N\overline{\kappa_{[1],m}} \\ &2\sum_{i=1<j}^{N} \left\langle \Delta k_{m.i} \Delta k_{m.j} \right\rangle \approx \overline{\kappa_{[2],m}} B_2\left(T_S, \tau_D\right) T^{-2} - N\overline{\kappa_{[2],m}} \end{aligned}. \tag{SI.13}$$

Inserting Eqs. (SI.5), (SI.12), and (SI.13) into Eq. (SI.11) results in an expectation value of the Q-estimator given by,

$$E\widehat{Q_m} = \frac{\overline{\kappa_{[2],m}} + \overline{\kappa_{[1],m}^2} - \overline{\kappa_{[1],m}}^2}{\overline{\kappa_{[1],m}}} - \frac{1}{N} - \frac{\overline{\kappa_{[2],m}} B_2\left(T_S, \tau_D\right)}{\overline{\kappa_{[1],m}} T_S^2}. \tag{SI.14}$$

We calculate the mean segmented Q-value from this equation by

$$\mathrm{MSQ}\left(T_S\right) = M^{-1} \sum_{m=1}^{M} \frac{\overline{\kappa_{[2],m}} + \overline{\kappa_{[1],m}^2} - \overline{\kappa_{[1],m}}^2}{\overline{\kappa_{[1],m}}} - M^{-1} \sum_{m=1}^{M} \frac{1}{N} - M^{-1} \sum_{m=1}^{M} \frac{\overline{\kappa_{[2],m}}}{\overline{\kappa_{[1],m}}} \frac{B_2\left(T_S, \tau_D\right)}{T_S^2}. \tag{SI.15}$$

Because the first term equals the MSQ function for photodepletion only (Eq. (SI.9)), we rewrite this equation in its final form,

$$\mathrm{MSQ}\left(T_S\right) = \mathrm{MSQ}_{PD}\left(T_S\right) - \frac{1}{N} - \mathrm{A}\left(Q_1, n\right)\frac{B_2\left(T_S, \tau_D\right)}{T_S^2}, \qquad \text{(SI.16)}$$

with the function $\mathrm{A}\left(Q_1, n\right)$ is given by $M^{-1}\sum_{m=1}^{M}\left(\overline{\kappa_{[2],m}}\big/\overline{\kappa_{[1],m}}\right)$. This equation is equivalent to Eq. 14 of the manuscript.

References:

1. Petersen KE, Petersen K (1989) Ergodic Theory. Cambridge University Press. 348 p.

2. Wu B, Müller JD (2005) Time-integrated fluorescence cumulant analysis in fluorescence fluctuation spectroscopy. Biophys J 89: 2721–2735. doi:10.1529/biophysj.105.063685.

3. Müller JD (2004) Cumulant analysis in fluorescence fluctuation spectroscopy. Biophys J 86: 3981–3992. doi:10.1529/biophysj.103.037887.

4. Hur K-H, Macdonald PJ, Berk S, Angert CI, Chen Y, et al. (2014) Quantitative Measurement of Brightness from Living Cells in the Presence of Photodepletion. PLoS ONE 9: e97440. doi:10.1371/journal.pone.0097440.
